# ﻿New genera and new species of Hahniidae (Araneae) from China, Laos, Myanmar, and Vietnam

**DOI:** 10.3897/zookeys.1187.112936

**Published:** 2023-12-20

**Authors:** Chang Chu, Yejie Lin, Shuqiang Li

**Affiliations:** 1 Institute of Zoology, Chinese Academy of Sciences, Beijing 100101, China Institute of Zoology, Chinese Academy of Sciences Beijing China; 2 University of Chinese Academy of Sciences, Beijing 100101, China University of Chinese Academy of Sciences Beijing China

**Keywords:** Comb-tailed spiders, diagnosis, endemic, Hahniinae, taxonomy

## Abstract

Four new genera and 11 new species of Hahniidae Bertkau, 1878 are described. The new genera are *Goblinia* Lin & Li, **gen. nov.**, with the type species *G.tiane* Lin & Li, **sp. nov.** (♂♀) from Guangxi, China; *Myahnia* Lin & Li, **gen. nov.**, with the type species *M.kanpetlet* Lin & Li, **sp. nov.** (♂♀) from Chin, Myanmar; *Troglohnia* Lin & Li, **gen. nov.**, with the type species *Tr.qiubei* Lin & Li, **sp. nov.** (♂♀) from Yunnan, China and *Typhlohnia* Lin & Li, **gen. nov.**, with the type species *Ty.rongshui* Lin & Li, **sp. nov.** (♂♀) from Guangxi, China. Seven additional new species are described: *Tr.dafang* Lin & Li, **sp. nov.** (♂♀) from Guizhou, China; *Tr.shidian* Lin & Li, **sp. nov.** (♀) from Yunnan, China; *Tr.wuding* Lin & Li, **sp. nov.** (♂♀) from Yunnan, China; *Ty.banlaksao* Lin & Li, **sp. nov.** (♀) from Bolikhamxay, Laos; *Ty.kaiyang* Lin & Li, **sp. nov.** (♀) from Guizhou, China; *Ty.sondoong* Lin & Li, **sp. nov.** (♂♀) from Quang Binh, Vietnam and *Ty.suiyang* Lin & Li, **sp. nov.** (♀) from Guizhou, China.

## ﻿Introduction

Asian spider taxonomists have published a large number of papers in the 21^st^ Century, but due to the rich biodiversity of the Southeast Asia fauna, there are still many unknown species (Li et al. 2021; Yang et al. 2021; Yao et al. 2021; Liu et al. 2022; Lu et al. 2022; Zhao et al. 2022; Zhang et al. 2023). Currently, 354 species in 23 genera of the spider family Hahniidae Bertkau, 1878, the so-called comb-tailed spiders, are known worldwide (WSC 2023), of which 53 species (45 endemic species) in China, followed by six species (four endemics) in Vietnam and one in Laos (WSC 2023). Hitherto, there have been no records from Myanmar (WSC 2023). In this paper, we report 11 new species and four new genera from China, Laos, Myanmar, and Vietnam.

## ﻿Material and methods

All specimens were preserved in 80% ethanol. The spermathecae were cleared in trypsin enzyme solution to dissolve non-chitinous tissues. Specimens were examined under a LEICA M205C stereomicroscope. Photomicrographs were taken with an Olympus C7070 zoom digital camera (7.1 megapixels). Photographs were stacked with Helicon Focus (v. 7.6.1) or Zerene Stacker (v. 1.04) and processed in Adobe Photoshop CC2022.

All measurements are in millimetres (mm) and were obtained with an Olympus SZX16 stereomicroscope with a Zongyuan CCD industrial camera. All measurements of body lengths do not include the chelicerae. Eye sizes are measured as the maximum diameter from either the dorsal or the frontal view. Legs were measured laterally. Leg measurements are given as follows: total length (femur, patella, tibia, metatarsus, tarsus). Four paratype males specimens (*Gobliniatiane* sp. nov., *Myahniakanpetlet* sp. nov., *Troglohniaqiubei* sp. nov. and *Typhlohniarongshui* sp. nov.) were used for electron microscopy. They were fragile after electron microscopy, so their variation data was not measured. The terminology used in the text and figures follows Zhang et al. (2011) and Huang et al. (2017).

Types from the current study are deposited in the Institute of Zoology, Chinese Academy of Sciences in Beijing (**IZCAS)**.

Abbreviations used in text: **AER** anterior eye row; **ALE** anterior lateral eye; **AME** anterior median eye; **C** conductor; **CD** copulatory duct; **CF** cymbial furrow; **CO** copulatory opening; **D** depression; **d** dorsal; **dRTA** dorsal retrolateral tibial apophysis; **E** embolus; **ET** embolic tooth; **FD** fertilization duct; **GA** glandular appendage; **H** hood; **MOA** median ocular area; **p** prolateral; **PA** patellar apophysis; **PER** posterior eye row; **PLE** posterior lateral eye; **PME** posterior median eye; **PS** primary spermatheca; **r** retrolateral; **RTA** retrolateral tibial apophysis; **S** spermatheca; **SD** sperm dust; **SS** secondary spermatheca; **v** ventral; **vRTA** ventral retrolateral tibial apophysis.

## ﻿Taxonomic account

### ﻿Family Hahniidae Bertkau, 1878

Ono and Ogata (2018) divided this family into two subfamilies: Hahniinae Bertkau, 1878 (type genus: *Hahnia* C. L. Koch, 1841) and Cicurininae F. O. Pickard-Cambridge, 1893 (type genus: *Cicurina* Menge, 1871).

### ﻿Subfamily Hahniinae Bertkau, 1878

#### ﻿Genus Goblinia

Taxon classificationAnimaliaAraneaeHahniidae

Lin & Li gen. nov.

18EA3FD4-14F1-5932-9069-C2180BC1C43F

https://zoobank.org/E1AF935B-398E-4BE9-A7E1-81798965C021

##### Type species.

*Gobliniatiane* sp. nov. from Guangxi, China.

##### Diagnosis.

*Goblinia* gen. nov. can be distinguished from *Iberina* Simon, 1881 by the spineless male palpal femur (Fig. 6A, B) [vs femur with 3–5 spines (see Růžička 2022: fig. 2O)], embolus shorter than perimeter of bulb (Figs 3A, 4A) [vs almost 2× longer (see Růžička 2022: fig. 4A–E)] and epigyne with one pair of spermathecae (Fig. 5B) [vs two pairs of spermathecae, except for *I.difficilis* (Harm, 1966) and *I.microphthalma* (Snazell & Duffey, 1980) (see Růžička 2022: fig. 5A–E)].

##### Description.

**Male.** Total length 1.87–2.40 (*n* = 5). Carapace pale yellow, covered with few black setae. PER longer than AER, AER and PER procurved. AME separated by less than their diameter, closer to ALE; PME separated by almost their diameter, approximately as far from ALE; Distance between AME and PME longer than that between ALE and PLE; ALE and PLE almost touching. Clypeus pale yellow, covered with few setae. Chelicerae pale yellow, with three promarginal and three retromarginal teeth, with granular stridulatory files retrolaterally (Fig. 2A). Endites, labium pale yellow, covered with few black setae. Sternum coloured as endites, covered with brown setae. Legs pale yellow. Opisthosoma oval, white. Spinnerets white, M-shaped in posterior view. Tracheal spiracle long and transverse, distance of spiracle to epigastric furrow as long as to spinnerets.

Palpal femur almost 4× longer than patella, spineless. Patella shorter than tibia. Retrolateral tibial apophysis almost as long as tibia, curved to almost 100° angle. Cymbium egg-shaped, cymbial furrow almost as long as bulb. Bulb discoid round, without conductor. Sperm duct with curved course. Embolus whip-shaped, starting at ca 4:30 o’clock position.

**Female.** Total length 1.96–2.55 (*n* = 5). Somatic characters as in male but chelicerae with two promarginal and three retromarginal teeth, stridulatory files absent.

Epigynal plate wider than long. Copulatory openings located anteriorly, round, touching each other. Copulatory ducts long and intertwined, beginning laminar. Glandular appendages round, touching each other. Spermathecae oval, located posteriorly, separated by less than radius.

##### Etymology.

The new generic name is a combination of *goblin* (a legendary creature that lives underground) and *Hahnia*. The gender is feminine.

##### Composition.

Currently monotypic: *Gobliniatiane* sp. nov.

##### Distribution.

China (Guangxi) (Fig. 30).

#### ﻿ Goblinia tiane

Taxon classificationAnimaliaAraneaeHahniidae

Lin & Li sp. nov.

5CFD5BC0-49A9-53E3-BD47-35BCE14F7645

https://zoobank.org/D311CBBE-BE0C-4137-A678-8AE138F6B480

**Figure 1.**
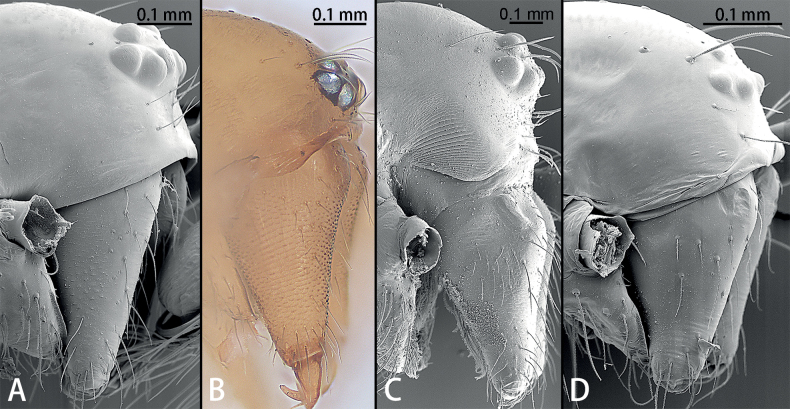
Cephalic region and chelicerae, paratype males, lateral view **A***Gobliniatiane* sp. nov. **B***Myahniakanpetlet* sp. nov. **C***Troglohniaqiubei* sp. nov. **D***Typhlohniarongshui* sp. nov.

**Figure 2.**
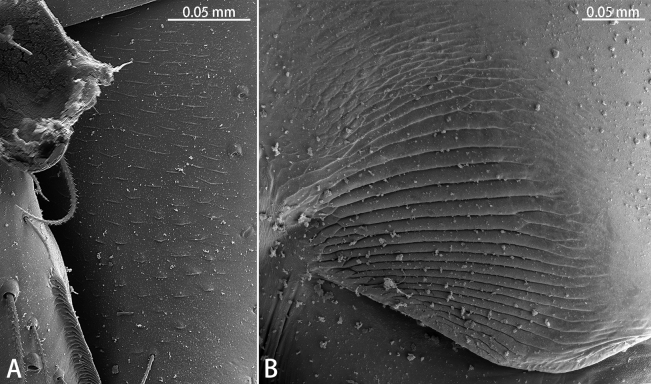
Stridulatory files, paratype males, lateral view **A***Gobliniatiane* sp. nov. **B***Troglohniaqiubei* sp. nov.

**Figure 3.**
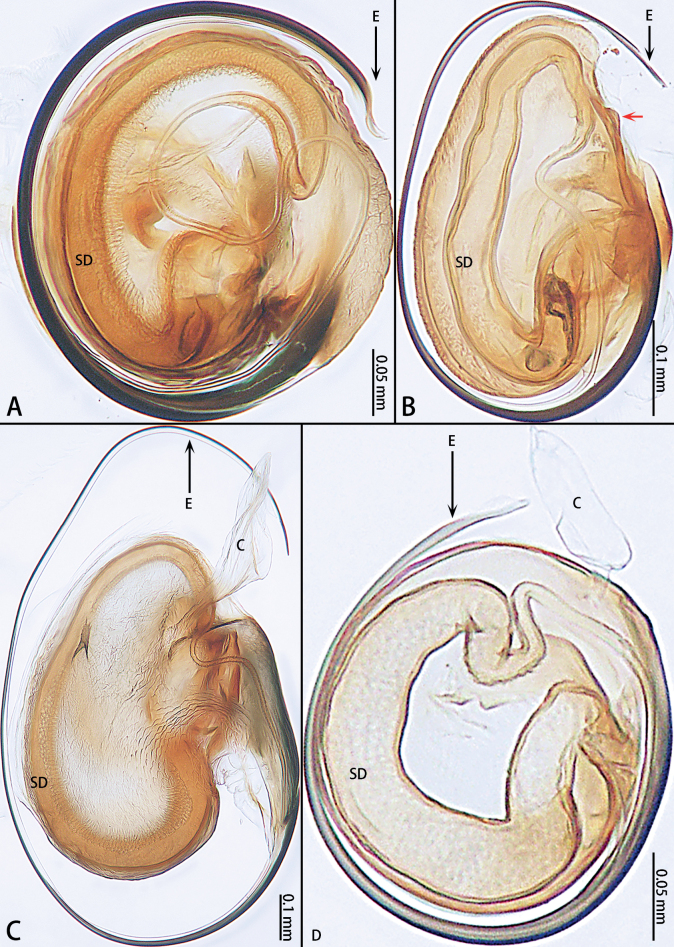
Paratype male bulbs, ventral view **A***Gobliniatiane* sp. nov. **B***Myahniakanpetlet* sp. nov. **C***Troglohniaqiubei* sp. nov. **D***Typhlohniarongshui* sp. nov. Red arrows show the tegular outgrowth. Abbreviations: C = conductor, E = embolus, SD = sperm duct.

**Figure 4.**
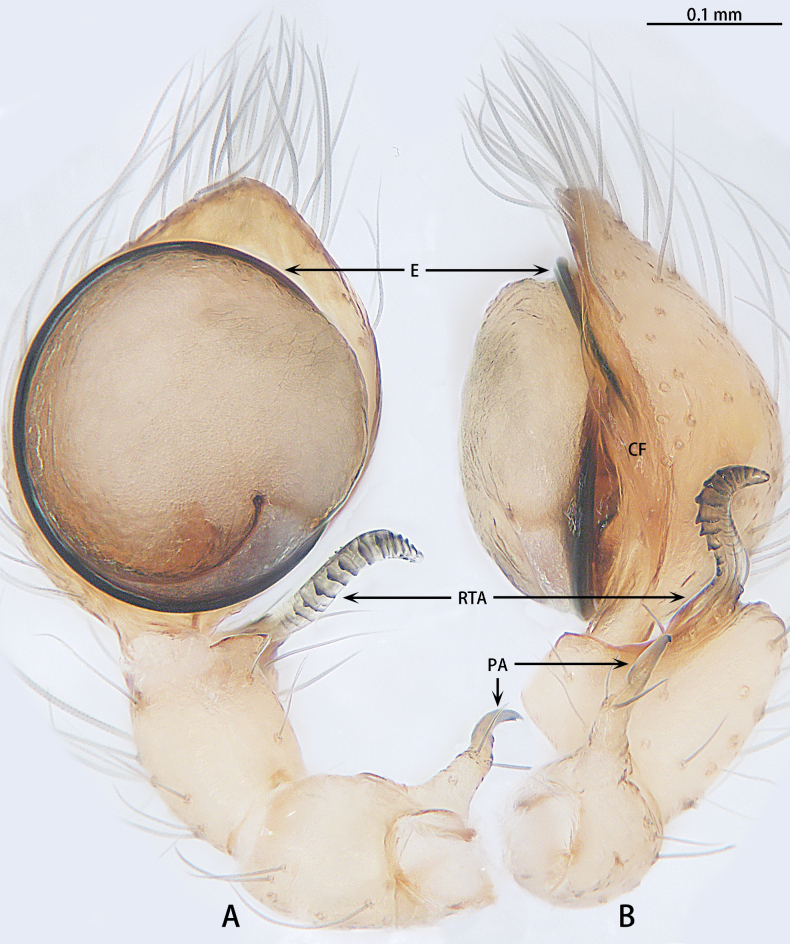
*Gobliniatiane* sp. nov., holotype male **A** ventral view **B** retrolateral view. Abbreviations: CF = cymbial furrow, E = embolus, PA = patellar apophysis, RTA = retrolateral tibial apophysis.

**Figure 5.**
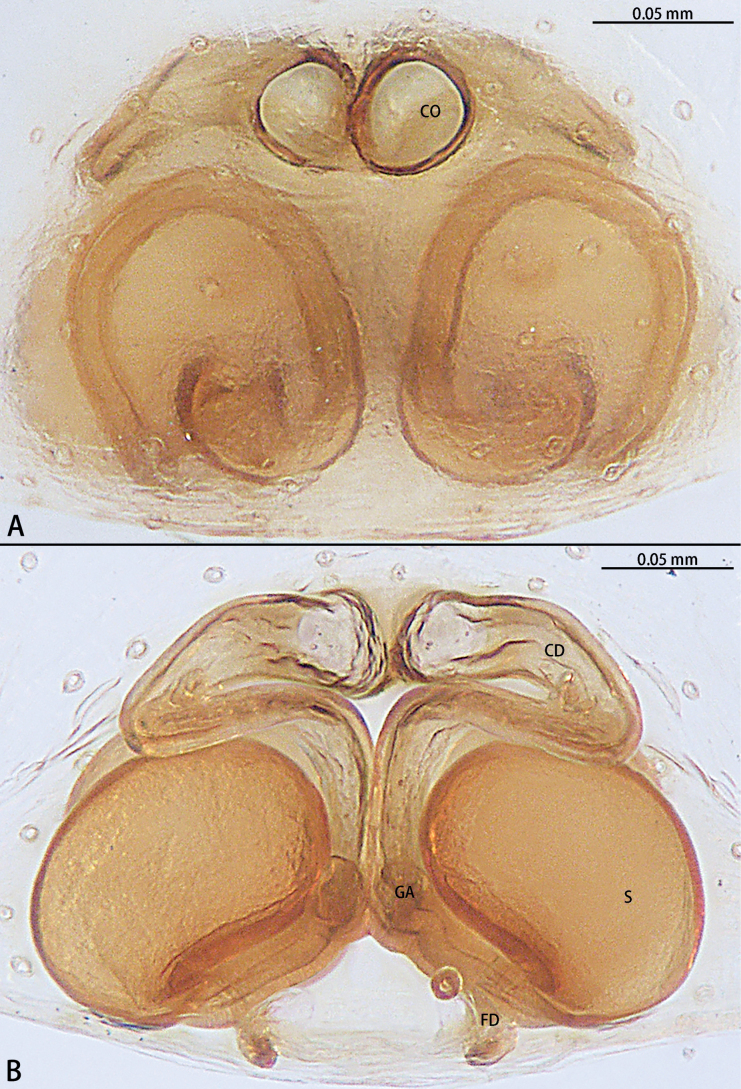
*Gobliniatiane* sp. nov., paratype female **A** epigyne, ventral view **B** vulva, dorsal view. Abbreviations: CD = copulatory duct, CO = copulatory opening, FD = fertilization duct, GA = glandular appendage, S = spermatheca.

**Figure 6.**
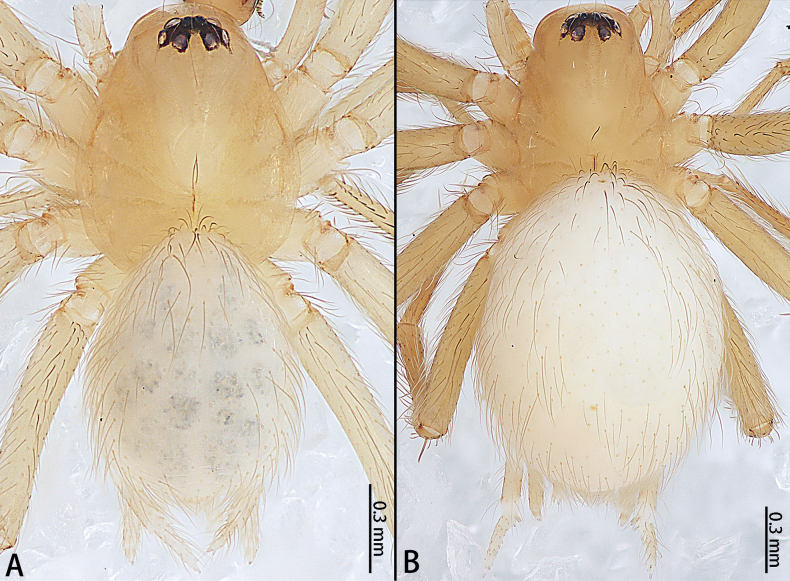
*Gobliniatiane* sp. nov., habitus, dorsal view **A** male holotype **B** female paratype.

Figs 1A , 2A , 3A , 4A, B , 5A, B , 6A, B , 30

##### Type material.

***Holotype***: ♂ (IZCAS-Ar44648), China, Guangxi: Hechi City, Tian’e County, Bala Town: No. 8 Cave, 24.9337°N, 107.0421°E, ca 685 m, 04.II.2015, Y. Li and Z. Chen leg. ***Paratypes***: 5♂ 5♀ (IZCAS-Ar44649–Ar44658), same data as holotype.

##### Diagnosis.

Same as for genus.

##### Description.

**Male** (holotype; Figs 4A, B, 6A). Total body length 1.87. Carapace 0.93 long, 0.74 wide; opisthosoma 0.94 long, 0.68 wide. AER and PER procurved slightly. Eye sizes and interdistances: AME 0.05, ALE 0.05, PME 0.05, PLE 0.05; AME–AME 0.01, AME–ALE 0.01, PME–PME 0.07, PME–PLE 0.03, ALE–PLE 0.02. MOA 0.12 long, front width 0.08, back width 0.15. Clypeus 0.14 high. Leg measurements: I 2.94 (0.85, 0.29, 0.64, 0.67, 0.49); II 2.75 (0.81, 0.28, 0.59, 0.60, 0.47); III 2.79 (0.77, 0.28, 0.60, 0.66, 0.48); IV 3.06 (0.93, 0.29, 0.73, 0.65, 0.46). Leg spination: patellae I–IV d1; tibiae I–II d1, III–IV p1 d1 r1; metatarsi III p1 d1 r1, IV p1 r1.

***Coloration*** (Fig. 6A). Carapace yellowish, with light brown radiating marks and indistinct marginal brown band. Fovea longitudinal, reddish-brown. Chelicerae, labium, gnathocoxae, and sternum yellowish. Legs yellowish. Opisthosoma oval, grey with dark pattern, venter grey without pattern. Spinnerets white.

***Palp*** (Fig. 4A, B). Patellar apophysis as long as patella, tip hook-shaped; patellar apophysis almost 1.5× longer than tip. Tibia with black, serrated retrolateral apophysis, S-shaped in retrolateral view. Cymbium almost egg-shaped, 1.2× longer than wide. Sperm duct long, basal thick part running around of tegulum, forming ~ 270° loop clockwise; middle thin part forming circular anticlockwise, diameter ~ 1/3 of bulb diameter; distal thin part running around of tegulum, forming circular clockwise. Embolus with wide, slightly membranous base and membranous, curved tip; its base arising at 4:30 o’clock position; tip hidden by cymbium in retrolateral view.

**Female** (paratype IZCAS-Ar44658; Figs 5A, B, 6B). Total body length 2.41. Carapace 0.97 long, 0.76 wide; opisthosoma 1.44 long, 1.09 wide. Eye sizes and interdistances: AME 0.04, ALE 0.07, PME 0.06, PLE 0.06; AME–AME 0.03, AME–ALE 0.01, PME–PME 0.07, PME–PLE 0.03, ALE–PLE 0.01. MOA 0.15 long, front width 0.09, back width 0.18. Clypeus 0.11 high. Leg measurements: I 2.42 (0.74, 0.31, 0.50, 0.49, 0.38); II 2.46 (0.73, 0.31, 0.48, 0.51, 0.43); III 2.58 (0.73, 0.31, 0.52, 0.57, 0.45); IV 3.25 (0.88, 0.32, 0.71, 0.76, 0.58). Leg spination: patellae I–IV d1; tibiae I–II d1, III–IV p1 d2 r1; metatarsi III p1 d1 r1, IV p1 r1 v1.

***Coloration*** (Fig. 6B). As in male but opisthosoma without pattern.

***Epigyne*** (Fig. 5A, B). Epigynal plate 1.35× wider than long. Copulatory openings located anteriorly, round, touching each other. Copulatory ducts intertwined, basal part with sharp twist, middle and distal part coiled twice around margin of spermathecae. Glandular appendages round, originating submedially to copulatory ducts, touching each other. Spermathecae oval, 4× wider than copulatory openings. Fertilization ducts directed at 11:00 o’clock position from spermathecae.

##### Variation.

Males (*n* = 4): total body length 1.89–2.40, carapace 0.87–1.09 long, 0.76–0.88 wide, opisthosoma 0.98–1.31 long, 0.71–0.98 wide. Females (*n* = 4): total body length 1.96–2.55, carapace 0.80–0.96 long, 0.63–0.77 wide, opisthosoma 1.09–1.59 long, 0.81–1.18 wide.

##### Etymology.

The specific epithet refers to the type locality; noun in apposition.

##### Distribution.

Known only from the type locality (Fig. 30).

#### ﻿Genus Myahnia

Taxon classificationAnimaliaAraneaeHahniidae

Lin & Li gen. nov.

4DFEECE5-9460-5F87-A17B-A5D6F964575C

https://zoobank.org/317A5532-7E8A-4393-9234-A1D857D1BD48

##### Type species.

*Myahniakanpetlet* sp. nov. from Chin, Myanmar.

##### Diagnosis.

**Figure 7.**
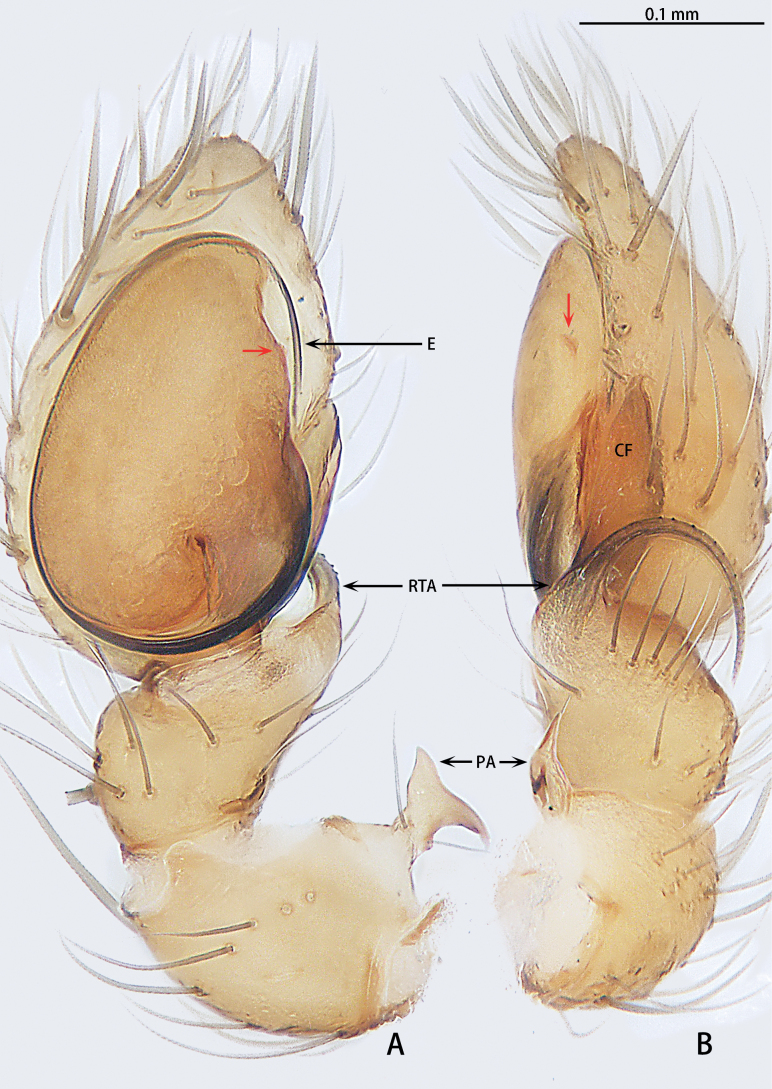
*Myahniakanpetlet* sp. nov., holotype male **A** ventral view **B** retrolateral view. Red arrows show the tegular outgrowth. Abbreviations: CF = cymbial furrow, E = embolus, PA = patellar apophysis, RTA = retrolateral tibial apophysis.

**Figure 8.**
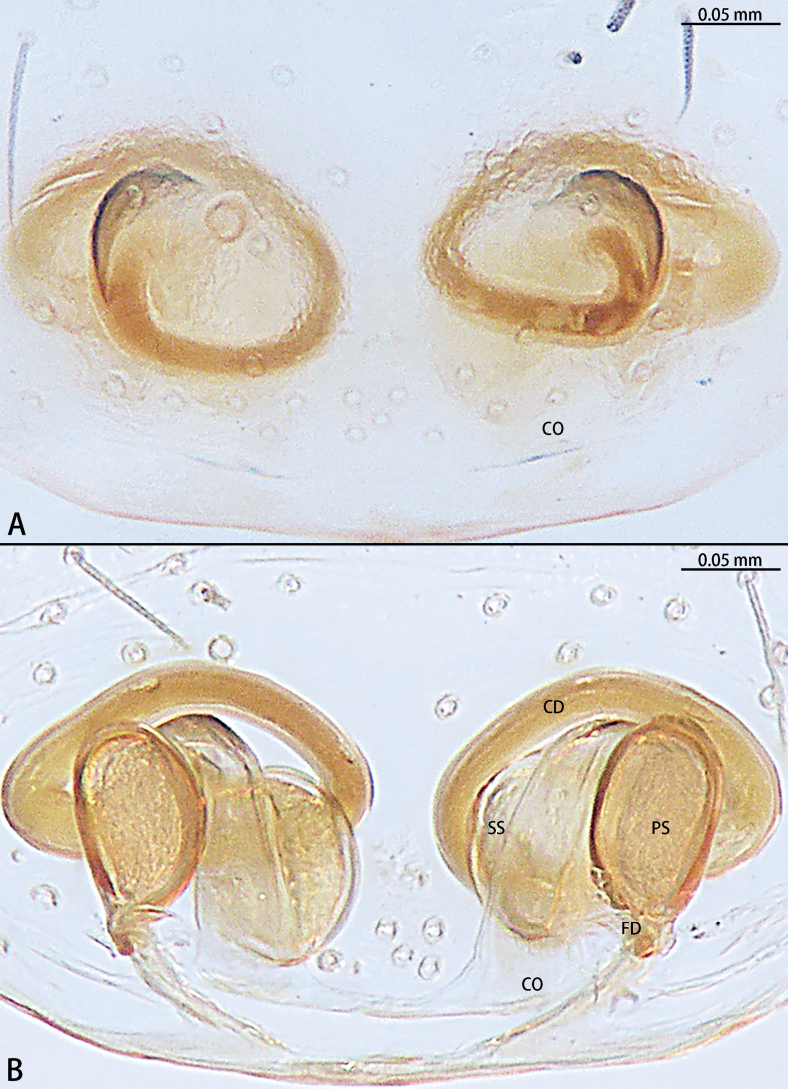
*Myahniakanpetlet* sp. nov., paratype female **A** epigyne, ventral view **B** vulva, dorsal view. Abbreviations: CD = copulatory duct, CO = copulatory opening, FD = fertilization duct, PS = primary spermatheca, SS = secondary spermatheca.

**Figure 9.**
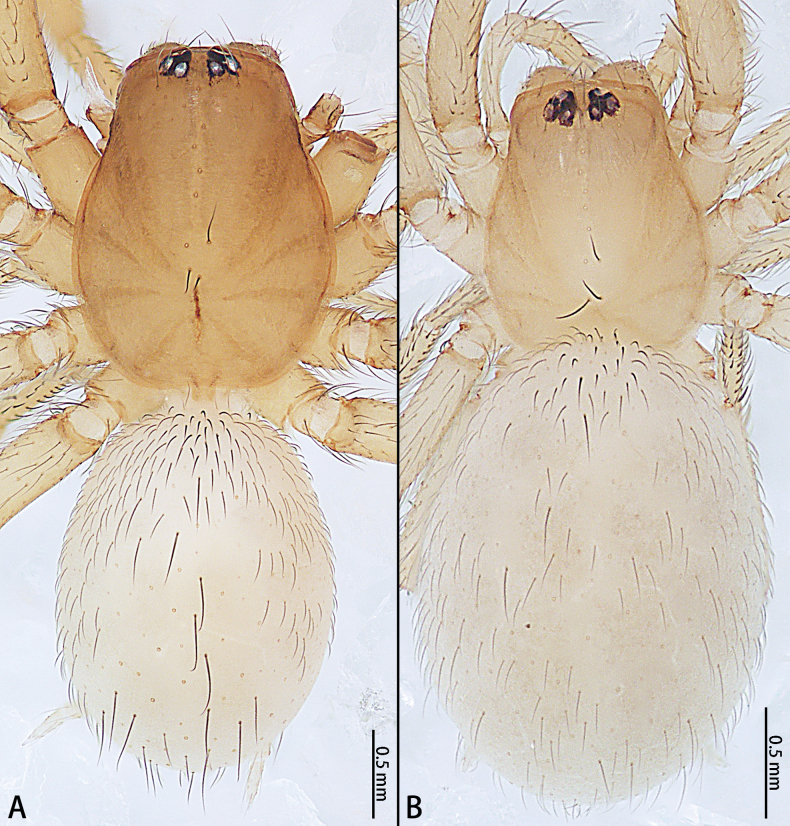
*Myahniakanpetlet* sp. nov., habitus, dorsal view **A** male holotype **B** female paratype.

*Myahnia* gen. nov. can be distinguished from *Hexamatia* Rivera-Quiroz, Petcharad & Miller, 2020 by the larger body size > 1.49 mm (Fig. 9A, B) [vs 1 mm (see Rivera-Quiroz et al. 2020: fig. 2a–c)], chelicerae with stridulatory files (Fig. 1B) (vs without), relatively longer cymbial furrow bend almost 0.5× length of cymbium (Fig. 7B) [vs almost 0.25× (see Rivera-Quiroz et al. 2020: fig. 3b)], conductor absent (Figs 3B, 7A) [vs present (see Rivera-Quiroz et al. 2020: fig. 3a, b, d, e)], sperm duct with U-shaped curve (Fig. 3B) [vs circular (see Rivera-Quiroz et al. 2020: fig. 3a, c)] and base of copulatory ducts membranous (Fig. 8B) [vs sclerotized (see Zhang et al. 2011: fig. 23E)].

##### Description.

**Male.** Small size. Carapace yellow, covered with few black setae. Six eyes in two rows; AME absent, PER longer than AER, PER procurved. ALE separated by almost their diameter; PME separated by longer than their diameter; ALE and PLE almost touching. Clypeus yellow, covered with several setae. Chelicerae yellow, with two promarginal and three retromarginal teeth, stridulatory files striped. Endites, labium yellow, covered with few black setae. Sternum coloured as endites, covered with brown setae. Legs yellow. Opisthosoma oval, grey without pattern. Spinnerets grey, straight in posterior view. Tracheal spiracle long and transverse, distance of spiracle to epigastric furrow as long as to spinnerets.

Palpal femur almost 3× longer than patella, spineless. Patella longer than tibia, with retroventral apophysis. Tibia with long, curved serrated retrolateral apophysis. Cymbium 1.7× longer than wide, cymbial furrow almost 0.5× longer than cymbium. Bulb oval, without conductor, with sclerotized apophysis retrolaterally. Embolus whip-shaped, starting at ca 3:00–5:00 o’clock position, curving clockwise along tegular margin.

**Female.** Total length 1.49–1.76 (*n* = 4). Somatic characters as in male but body pale yellow and stridulatory files absent.

Epigynal plate 2× wider than long. Copulatory openings located posteriorly, unobvious. Copulatory ducts curved, basal part wide and laminar. Vulva with two pairs of spermathecae. Fertilization ducts small, sickle-shaped.

##### Etymology.

The new generic name is a combination of *Myanmar* and *Hahnia*. The gender is feminine.

##### Composition.

Currently monotypic: *Myahniakanpetlet* sp. nov.

##### Distribution.

Myanmar (Fig. 30).

#### ﻿ Myahnia kanpetlet

Taxon classificationAnimaliaAraneaeHahniidae

Lin & Li sp. nov.

BDA1FB36-1B0C-5CA7-A4C8-8F34C7CB121F

https://zoobank.org/4C1D6538-FBA1-405C-8B59-0D1AF5ACD45F

Figs 1B , 3B , 7A, B , 8A, B , 9A, B , 30

##### Type material.

***Holotype***: ♂ (IZCAS-Ar44659), Myanmar, Chin State: Near 16.5 km of the roadside between Kanpetlet to Nat Ma Taung National Park, 21.2199°N, 93.2687°E, ca 2789 m, 30.IV.2017, Y. Li and Z. Chen leg. ***Paratypes***: 1♂ 4♀ (IZCAS-Ar44660–Ar44664), same data as holotype.

##### Diagnosis.

Same as for genus.

##### Description.

**Male** (holotype; Figs 7A, B, 9A). Total body length 2.15. Carapace 1.02 long, 0.77 wide; opisthosoma 1.13 long, 0.81 wide. PER procurved slightly. Eye sizes and interdistances: ALE 0.05, PME 0.04, PLE 0.05; PME–PME 0.06, PME–PLE 0.01, ALE–PLE 0.01. Clypeus 0.03 high. Leg measurements: I 2.75 (0.85, 0.34, 0.69, 0.51, 0.36); II 2.37 (0.75, 0.33, 0.52, 0.44, 0.33); III 1.98 (0.59, 0.28, 0.36, 0.44, 0.31); IV 2.47 (0.77, 0.31, 0.56, 0.45, 0.38). Leg spination: femora I–II d1; patellae I–IV d1; tibiae III p1 d1 r1 v2, IV p1 r2 v2; metatarsi III p1 d1 r1, IV p1 r1.

***Coloration*** (Fig. 9A). Carapace yellow, with dark yellow radial grooves. Fovea reddish-brown. Chelicerae, labium and gnathocoxae yellow; sternum yellowish. Sternum without markings. Legs yellow. Opisthosoma oval, grey. Spinnerets grey.

***Palp*** (Fig. 7A, B). Patellar apophysis tip widening; distal part wider that apophysis length. Retrolateral tibial apophysis curved and serrated, almost as long as tibia, C-shaped in retrolateral view. Cymbium elongate egg-shaped, ca 1.7× longer than wide, cymbial furrow almost 2× shorter than cymbium. Bulb oval. Conductor absent, but tegular outgrowth present, sclerotized, located retrolaterally (arrowed in Figs 3B, 7A, B). Middle of sperm duct bent in U-shape. Embolus slender and whip-shaped; base of embolus arising at 3:00–5:00 o’clock position, clockwise, makes ~ 360° loop.

**Female** (paratype IZCAS-Ar44664; Figs 8A, B, 9B). Total body length 1.76. Carapace 0.69 long, 0.50 wide; opisthosoma 1.07 long, 0.78 wide. Eye sizes and interdistances: ALE 0.03, PME 0.01, PLE 0.02; PME–PME 0.05, PME–PLE 0.01, ALE–PLE 0.02. Clypeus 0.02 high. Leg measurements: I 1.56 (0.48, 0.20, 0.35, 0.28, 0.25); II 1.38 (0.42, 0.19, 0.28, 0.25, 0.24); III 1.24 (0.37, 0.18, 0.22, 0.26, 0.21); IV 1.62 (0.48, 0.20, 0.34, 0.34, 0.26). Leg spination: femora I–III d1; patellae I–IV d1; tibiae III–IV p1 d1 r1 v1; metatarsi III–IV p1 d1 r1.

***Coloration*** (Fig. 9B). As in male but body pale yellow.

***Epigyne*** (Fig. 8A, B). Copulatory openings large, ~ 4× shorter than epigyne width, located on posterior edge. Copulatory ducts intertwined, shorter part with twist, longer part with two twists. Secondary spermathecae oval, almost as long as primary spermatheca. Primary spermathecae elongate. Fertilization ducts directed at 10:00 o’clock position from spermathecae.

##### Variation.

Females (*n* = 3): total body length 1.49–1.76, carapace 0.68–0.71 long, 0.50–0.52 wide, opisthosoma 0.81–1.05 long, 0.60–0.75 wide.

##### Etymology.

The specific epithet refers to the type locality; noun in apposition.

##### Distribution.

Known only from the type locality (Fig. 30).

#### ﻿Genus Troglohnia

Taxon classificationAnimaliaAraneaeHahniidae

Lin & Li gen. nov.

28117E71-5F0C-5684-BF47-3F94EAC2ED24

https://zoobank.org/1D333387-6E69-4CCB-862E-256EE2EECB15

##### Type species.

*Troglohniaqiubei* sp. nov. from Yunnan, China.

##### Diagnosis.

**Figure 10.**
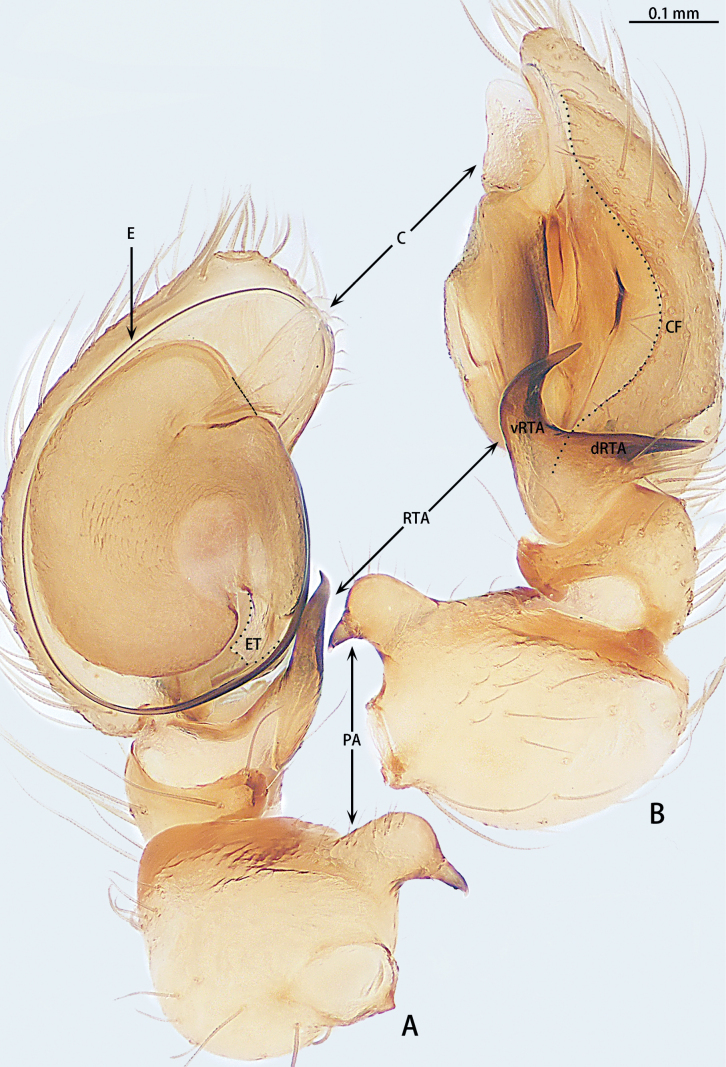
*Troglohniadafang* sp. nov., holotype male **A** ventral view **B** retrolateral view. Dashed line shows conductor stalk. Abbreviations: C = conductor, CF = cymbial furrow, dRTA = dorsal retrolateral tibial apophysis, E = embolus, ET = embolic tooth, PA = patellar apophysis, RTA = retrolateral tibial apophysis, vRTA = ventral retrolateral tibial apophysis.

*Troglohnia* gen. nov. can be distinguished from all other genera of Hahniidae by having stridulatory files on sides of pars cephalica (Figs 1C, 2B), strongly modified patella 1.5–2× wider than tibia (Figs 10A, 12A, 15A), retrolateral tibial apophysis with two arms (Figs 10B, 12B, 15B), edge of cymbial furrow S-shaped (Figs 10B, 12B, 15B), base of embolus with tooth and embolus running along tegulum, on some distance, basal part rises from tegular bend at 90° (Figs 10A, 12A, 15A), genital groove with a pair of hoods posteriorly, distance between hoods almost 1.5–2× longer than length of epigynal plate (Fig. 18A–D), and copulatory ducts with a fork at the expanded part of them (Fig. 18A–D).

##### Description.

**Male.** Total length 2.33–3.21 (*n* = 8). Carapace yellowish, middle region with indistinct brown band, margin with brown pattern, lateral cephalic region with stridulatory files. PER longer than AER, AER straight, PER procurved. AME separated by less than diameter; PME separated by almost diameter, approximately as far from ALE; distance between AME and PME longer than that between ALE and PLE; ALE and PLE almost touching. Clypeus pale yellow, covered with several setae. Chelicerae pale yellow, with two or three promarginal and three or four retromarginal teeth, chelicerae with stridulatory files. Endites, labium pale yellow, covered with few black setae. Sternum brown, covered with brown setae. Legs pale yellow. Opisthosoma grey, middle of anteriorly and laterally with rod-shaped brown patterns, middle of posteriorly with inverted V-shaped brown patterns; venter with brown patterns and brown ring around spinnerets. Spinnerets base brown and tip white, straight in posterior view. Tracheal spiracle long and transverse, located at 3/4 of opisthosoma length.

Palpal femur almost 2× longer than patella, spineless. Patella modified, strongly swollen, longer and > 1.5× wider than tibia. Retrolateral tibial apophysis with 2 sickle-shaped and without serrated arms, basal with an apophysis with a line of setae. Cymbium kidney-shaped, ~ 1.5× longer than wide. Cymbial furrow S-shaped, almost as long as cymbium. Bulb almost oval, ca 1.23× longer than wide. Sperm duct with U-shaped curve (Fig. 3C). Conductor almost 0.5× longer than bulb, length of stalk almost 1/3–1/2 of total conductor length. Embolic base wide, with embolic tooth. Embolus starting at ca 1:30–4:00 o’clock from tegular, curving clockwise, embolic tip covered by bulb.

**Female.** Total length 2.25–3.86 (*n* = 21). Somatic characters as in male but chelicerae with three promarginal and three or four retromarginal teeth, stridulatory files absent.

Epigynal plate almost 1.3–1.45× wider than long, genital groove with a pair of posterior hoods. Copulatory openings located medium, arc-shaped. Copulatory ducts long, strongly convoluted, base thick, bifurcate, then become thinner; short one connected to secondary spermathecae, other connected to primary spermathecae. Primary spermathecae oval or bean-shaped, secondary spermathecae globular. Fertilization ducts laminar, sickle-shaped.

##### Etymology.

The new generic name is a combination of *Troglobiont* (refers to the cave habitat) and *Hahnia*. The gender is feminine.

##### Composition.

This new genus includes four species: *Troglohniadafang* sp. nov. (♂♀), *T.qiubei* sp. nov. (♂♀), *T.shidian* sp. nov. (♀), and *T.wuding* sp. nov. (♂♀).

##### Distribution.

China (Guizhou, Yunnan) (Fig. 30).

#### ﻿ Troglohnia dafang

Taxon classificationAnimaliaAraneaeHahniidae

Lin & Li sp. nov.

913B9777-F4FD-534B-AFAC-69DB3D7587A6

https://zoobank.org/5D13FD4B-1068-464F-8E04-6373ACEEB637

**Figure 11.**
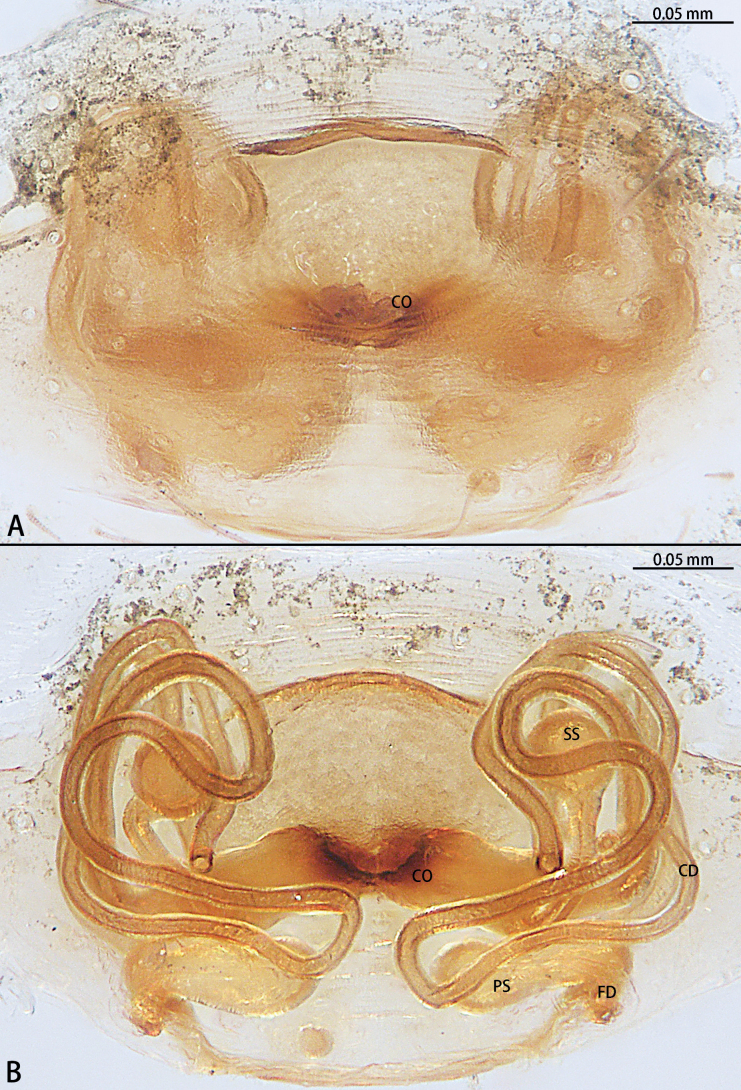
*Troglohniadafang* sp. nov., paratype female **A** epigyne, ventral view **B** vulva, dorsal view. Abbreviations: CD = copulatory duct, CO = copulatory opening, FD = fertilization duct, PS = primary spermatheca, SS = secondary spermatheca.

Figs 10A, B , 11A, B , 17A , 18A , 19A , 20A, B , 30

##### Type material.

***Holotype***: ♂ (IZCAS-Ar44666), China, Guizhou: Dafang County, Sanhe Villiage, Yelaoda Cave, 27.1817°N, 105.4713°E, ca 1438 m, 03.V.2007, Y. Li and J. Liu leg. ***Paratypes***: 1♂ 4♀ (IZCAS-Ar44667–Ar44671), same data as holotype.

##### Diagnosis.

**Figure 12.**
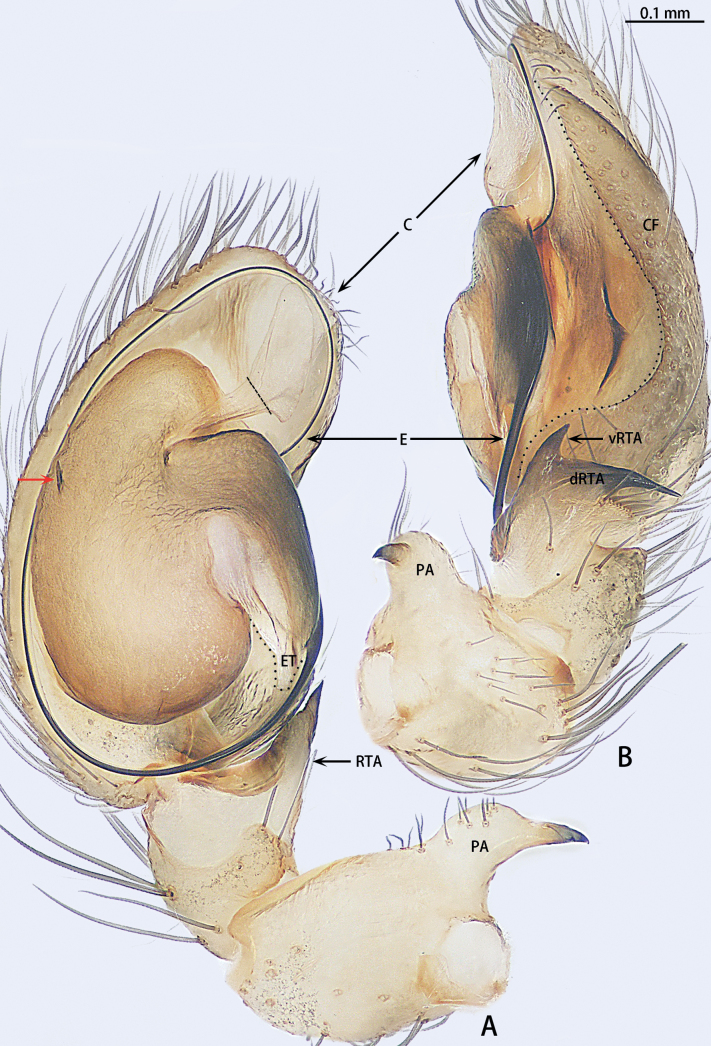
*Troglohniaqiubei* sp. nov., holotype male **A** ventral view **B** retrolateral view. Dashed line shows conductor stalk; red arrow shows the process on tegulum. Abbreviations: C = conductor, CF = cymbial furrow, dRTA = dorsal retrolateral tibial apophysis, E = embolus, ET = embolic tooth, PA = patellar apophysis, RTA = retrolateral tibial apophysis, vRTA = ventral retrolateral tibial apophysis.

**Figure 13.**
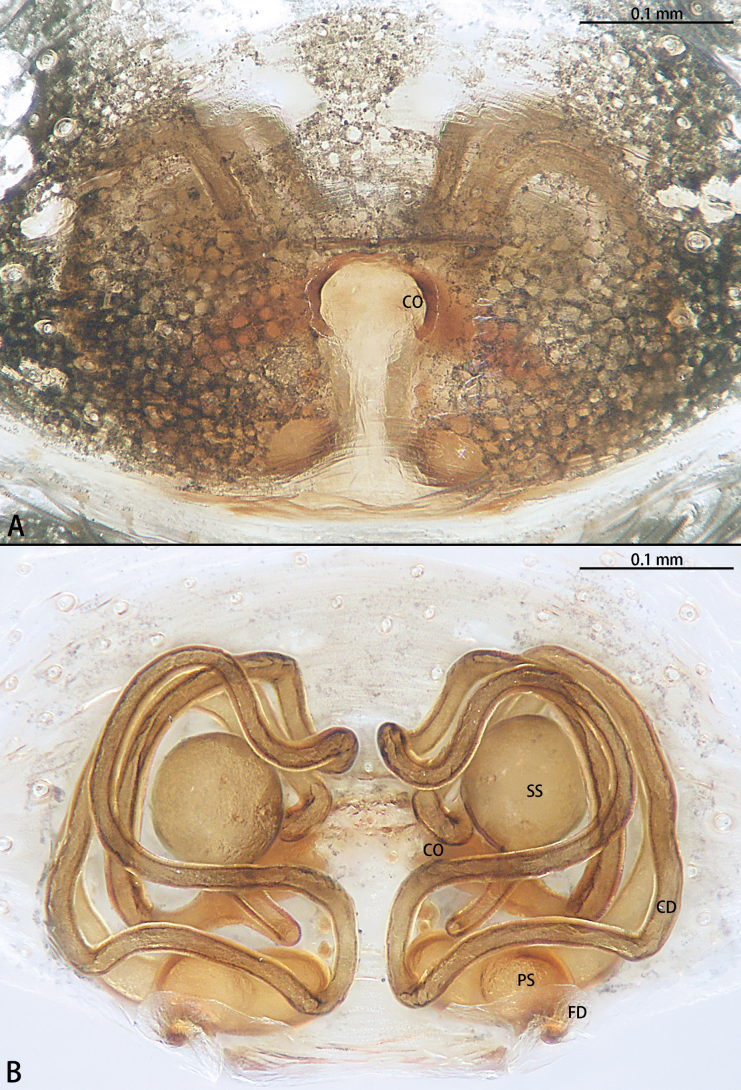
*Troglohniaqiubei* sp. nov., paratype female **A** epigyne, ventral view **B** vulva, dorsal view. Abbreviations: CD = copulatory duct, CO = copulatory opening, FD = fertilization duct, PS = primary spermatheca, SS = secondary spermatheca.

**Figure 14.**
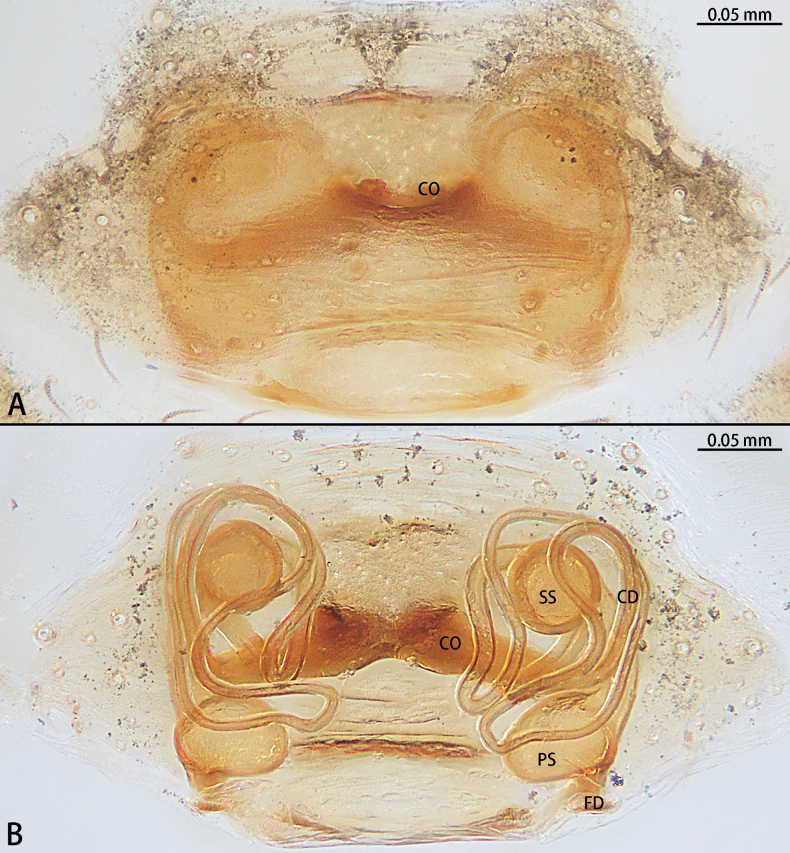
*Troglohniashidian* sp. nov., holotype female **A** epigyne, ventral view **B** vulva, dorsal view. Abbreviations: CD = copulatory duct, CO = copulatory opening, FD = fertilization duct, PS = primary spermatheca, SS = secondary spermatheca.

*Troglohniadafang* sp. nov. can be distinguished from *T.qiubei* sp. nov. by the tip of patellar apophysis pointed to 9:30 o’clock position (Fig. 17A) [vs 10:30 o’clock position (Fig. 17B)], ventral retrolateral tibial apophysis almost as long as dorsal retrolateral tibial apophysis (Fig. 10B) [vs 1:2 (Fig. 12B)], conductor stalk makes up 1/3 of total conductor length (Fig. 10A) [vs 1/2 (Fig. 12A)], process on tegulum absent (Fig. 10A) [vs present (arrowed in Fig. 12A)], copulatory openings touching each other (Fig. 11A) [vs facing each other (Fig. 13A)], diameter of primary spermathecae ~ 2× diameters of secondary spermathecae (Fig. 11B) [vs 1.5× (Fig. 13B)], distance between primary spermathecae and secondary spermathecae ~ 2× diameters of secondary spermathecae (Fig. 11B) [vs 1× (Fig. 13B)], secondary spermathecae separated by ~ 4× diameters (Fig. 11B) [vs 1.5× (Fig. 13B)], and fertilization ducts pointing to 9:00 o’clock position (Fig. 11B) [vs 7:30 o’clock position (Fig. 13B)]. Females of *T.dafang* sp. nov. can be distinguished from those of *T.shidian* sp. nov. by the ratio of diameter of secondary spermathecae to length of branched shorter copulatory ducts almost 1:2 (Fig. 11B) [vs 1:1 (Fig. 14B)], primary spermathecae elongate bean-shaped, separated by less than one diameter (Fig. 11B) [vs oval, separated by more than 2× diameters (Fig. 14B)], diameter of primary spermathecae ~ 2× diameters of secondary spermathecae (Fig. 11B) [vs 1.2× (Fig. 14B)], distance between primary spermathecae and secondary spermathecae ~ 2× diameters of secondary spermathecae (Fig. 11B) [vs 1× (Fig. 14B)] and secondary spermathecae separated by ~ 4× diameters (Fig. 11B) [vs 3× (Fig. 14B)].

##### Description.

**Male** (holotype; Figs 10A, B, 17A, 20A). Total body length 2.47. Carapace 1.18 long, 0.97 wide; opisthosoma 1.29 long, 0.94 wide. Eye sizes and interdistances: AME 0.05, ALE 0.08, PME 0.07, PLE 0.06; AME–AME 0.03, AME–ALE 0.02, PME–PME 0.09, PME–PLE 0.04, ALE–PLE 0.03. MOA 0.16 long, front width 0.10, back width 0.19. Clypeus 0.18 high. Chelicerae with three promarginal and four retromarginal teeth. Leg measurements: I 4.09 (1.18, 0.41, 0.96, 0.86, 0.68); II 3.88 (1.11, 0.41, 0.86, 0.83, 0.67); III 3.44 (0.93, 0.33, 0.75, 0.81, 0.62); IV 4.35 (1.15, 0.39, 1.02, 1.08, 0.71). Leg spination: patella III d1; tibiae III p1 d1 v1, IV p1 d1 r1 v1.

***Coloration*** (Fig. 20A). Carapace yellowish, middle region with indistinct brown band, margin with brown pattern. Fovea reddish-brown. Chelicerae, labium, gnathocoxae, and sternum yellowish. Sternum with dark marking. Legs yellowish. Opisthosoma oval, grey, middle of anteriorly and laterally with rod-shaped brown patterns, middle of posteriorly with inverted V-shaped brown patterns; venter with brown patterns and brown ring around spinnerets. Spinnerets base brown and tip white.

***Palp*** (Figs 10A, B, 17A). Patellar apophysis with wide base and narrowed tip, the narrowed tip shorter than the wide base. Ventral retrolateral tibial apophysis curved, almost as long as dorsal retrolateral tibial apophysis, but wider. Cymbium 1.5× longer than wide. Cymbial furrow almost as long as cymbium. Bulb almost oval. Medium of tegulum with globular membranous area. Length of stalk of conductor almost 1/3 of total conductor length. Embolic tooth terminal flat. Embolus slender and whip-shaped.

**Female** (paratype IZCAS-Ar44671; Figs 11A, B, 18A, 19A, 20B). Total body length 2.41. Carapace 0.92 long, 0.69 wide; opisthosoma 1.50 long, 1.16 wide. Eye sizes and interdistances: AME 0.03, ALE 0.09, PME 0.08, PLE 0.09; AME–AME 0.01, AME–ALE 0.02, PME–PME 0.09, PME–PLE 0.04, ALE–PLE 0.01. MOA 0.18 long, front width 0.10, back width 0.21. Clypeus 0.17 high. Chelicerae with three promarginal and three retromarginal teeth. Leg measurements: I 3.35 (0.99, 0.37, 0.75, 0.70, 0.54); II 3.17 (0.91, 0.36, 0.69, 0.68, 0.53); III 3.04 (0.89, 0.34, 0.63, 0.70, 0.48); IV 3.85 (1.07, 0.37, 0.93, 0.91, 0.57). Leg spination: femora I–III d1; patellae III–IV d1; tibiae III p1 d1 v1, IV p1 d1 r1 v2; metatarsi III d1 v2, IV p1 v2.

***Coloration*** (Fig. 20B). As in male but body yellow.

***Epigyne*** (Figs 11A, B, 18A, 19A). Epigynal plate 1.3× wider than long. Hoods 3× deeper than wide. The posterior edge of copulatory openings touching, slightly curved. The width of thick copulatory ducts base 2× wider than the thinner part. The branched shorter copulatory ducts connected to the secondary spermathecae, the other connected to the primary spermathecae. Primary spermathecae elongate bean-shaped, 2× wider than the secondary spermathecae. Fertilization ducts directed at 9:00 o’clock position from spermathecae.

##### Variation.

Male: total body length 2.33, carapace 1.15 long, 0.93 wide, opisthosoma 1.18 long, 0.92 wide. Females (*n* = 3): total body length 2.25–2.88, carapace 1.00–1.14 long, 0.73–0.88 wide, opisthosoma 1.23–1.74 long, 0.86–1.30 wide.

##### Etymology.

The specific epithet refers to the type locality; noun in apposition.

##### Distribution.

Known only from the type locality (Fig. 30).

#### ﻿ Troglohnia qiubei

Taxon classificationAnimaliaAraneaeHahniidae

Lin & Li sp. nov.

1ADEA9B9-AAD3-5625-8EFA-3FDD2473C031

https://zoobank.org/ECD0EC6E-BD4B-41F3-931F-A49F6E2F589F

Figs 1C , 2B , 3C , 12A, B , 13A, B , 17B , 18B , 19B , 20C, D , 30

##### Type material.

***Holotype***: ♂ (IZCAS-Ar44672), China, Yunnan: Wenshan Zhuang and Miao Autonomous Pref., Qiubei County, Shuanglongying Town, Puzhehei Villiage, Dongjiadashi Cave, 24.1422°N, 104.0990°E, ca 1455 m, 19.VIII.2010, Z. Yao, X. Wang and C. Wu leg. ***Paratypes***: 5♂ 5♀ (IZCAS-Ar44673–Ar44682), same data as holotype.

##### Diagnosis.

**Figure 15.**
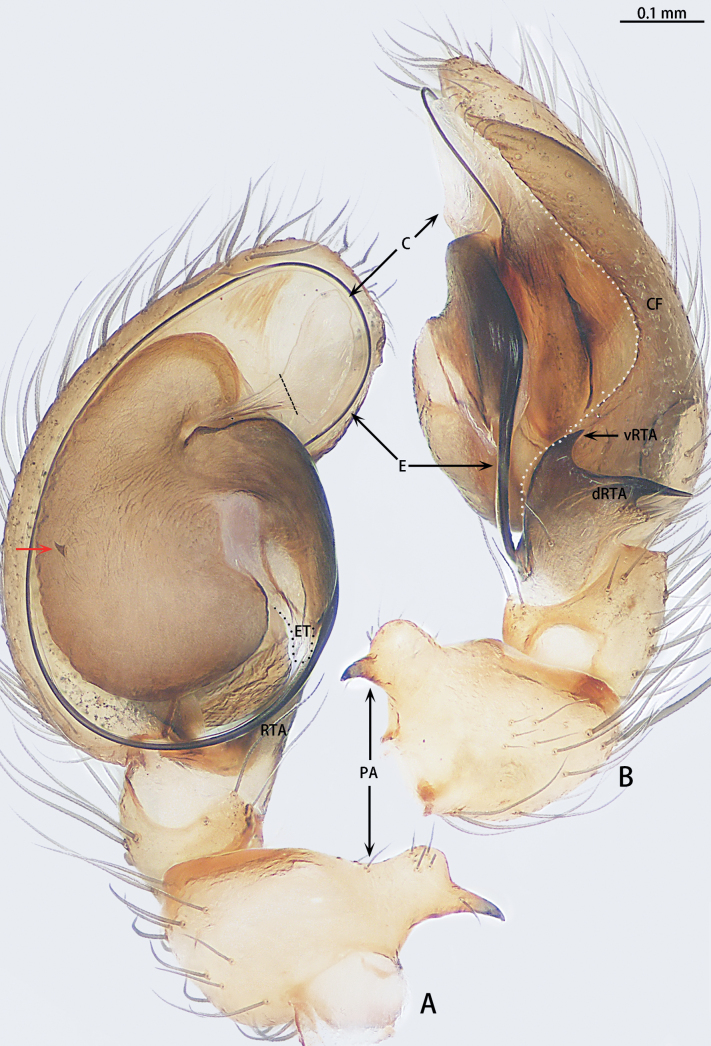
*Troglohniawuding* sp. nov., holotype male **A** ventral view **B** retrolateral view. Dashed line shows conductor stalk; red arrow shows the process on tegulum. Abbreviations: C = conductor, CF = cymbial furrow, dRTA = dorsal retrolateral tibial apophysis, E = embolus, ET = embolic tooth, PA = patellar apophysis, RTA = retrolateral tibial apophysis, vRTA = ventral retrolateral tibial apophysis.

**Figure 16.**
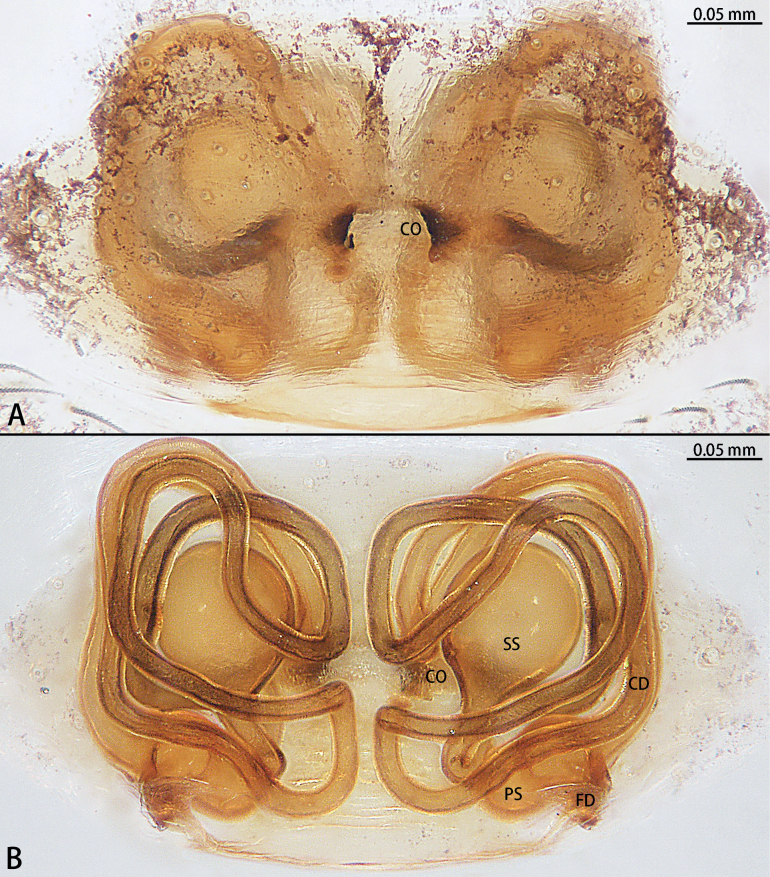
*Troglohniawuding* sp. nov., paratype female **A** epigyne, ventral view **B** vulva, dorsal view. Abbreviations: CD = copulatory duct, CO = copulatory opening, FD = fertilization duct, PS = primary spermatheca, SS = secondary spermatheca.

**Figure 17.**
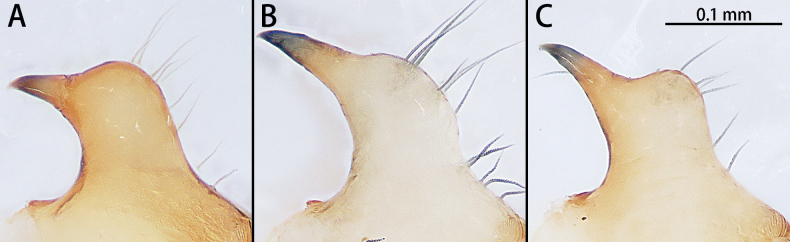
Patella apophyses of *Troglohnia* gen. nov., lateral view **A***Tr.dafang* sp. nov. **B***Tr.qiubei* sp. nov. **C***Tr.wuding* sp. nov.

*Troglohniaqiubei* sp. nov. can be distinguished from *T.wuding* sp. nov. by the male ventral retrolateral tibial apophysis almost straight and as wide as dorsal retrolateral tibial apophysis (Fig. 12B) [vs strongly curved and wider than dorsal retrolateral tibial apophysis (Fig. 15B)], embolic tooth terminal flat (Fig. 12A) [vs terminal finger-shaped (Fig. 15A)], process on anterior 1/3 of tegulum (arrowed in Fig. 12A) [vs on anterior 3/5 (arrowed in Fig. 15A)] and female can be distinguished by the diameter of secondary spermathecae less than diameter of primary spermathecae (Fig. 13B) [vs almost same diameter (Fig. 16B)], distance between primary spermathecae and secondary spermathecae more than half of diameter of secondary spermathecae (Fig. 13B) [vs less than half of diameter (Fig. 16B)], secondary spermathecae separated by ~ 1.5× diameters (Fig. 13B) [vs 1× (Fig. 16B)] and fertilization ducts pointing to 7:30 o’clock position (Fig. 13B) [vs 9:00 o’clock position (Fig. 16B)]. *T.qiubei* sp. nov. also resembles *T.dafang* sp. nov., but can be distinguished by the tip of patellar apophysis pointed to 10:30 o’clock position (Fig. 17B) [vs 9:30 o’clock position (Fig. 17A)], ventral retrolateral tibial apophysis shorter than dorsal retrolateral tibial apophysis (Fig. 12B) [vs almost as long as dorsal retrolateral tibial apophysis (Fig. 10B)], conductor stalk makes up 1/2 of total conductor length (Fig. 12A) [vs 1/3 (Fig. 10A)], process on tegulum present (Fig. 12A) [vs absent (Fig. 10A)], copulatory openings facing each other (Fig. 13A) [vs touching each other (Fig. 11A)], diameter of primary spermathecae ~ 1.5× diameters of secondary spermathecae (Fig. 13B) [vs 2× (Fig. 11B)], distance between primary spermathecae and secondary spermathecae ~ 1× diameter of secondary spermathecae (Fig. 13B) [vs 2× (Fig. 11B)], secondary spermathecae separated by ~ 1.5× diameters (Fig. 13B) [vs 4× (Fig. 11B)], and fertilization ducts pointing to 7:30 o’clock position (Fig. 13B) [vs 9:00 o’clock position (Fig. 11B)].

##### Description.

**Male** (holotype; Figs 12A, B, 17B, 20C). Total body length 2.92. Carapace 1.34 long, 1.05 wide; opisthosoma 1.58 long, 1.16 wide. AER straight and PER procurved slightly. Eye sizes and interdistances: AME 0.04, ALE 0.09, PME 0.08, PLE 0.09; AME–AME 0.05, AME–ALE 0.01, PME–PME 0.08, PME–PLE 0.04, ALE–PLE 0.02. MOA 0.20 long, front width 0.11, back width 0.24. Clypeus 0.17 high. Chelicerae with three promarginal and four retromarginal teeth. Leg measurements: I 4.86 (1.39, 0.44, 1.12, 1.05, 0.86); II 4.64 (1.30, 0.45, 1.03, 1.04, 0.82); III 4.29 (1.18, 0.41, 0.95, 1.02, 0.73); IV 5.27 (1.40, 0.44, 1.21, 1.34, 0.88). Leg supination: femora I–III d1; tibiae III v2, IV d1 r1 v1; metatarsi III p1 v2, IV p1 r1 v2.

***Coloration*** (Fig. 20C). Carapace yellowish, with indistinct dark yellow radial grooves, middle region with shield-shaped brown band, margin with brown pattern. Fovea longitudinal, reddish-brown. Ocular area with slight brown band; eight eyes without distinct black rims. Chelicerae, labium, gnathocoxae, and sternum yellowish. Sternum with brown marking and lighter heart region. Legs yellowish. Opisthosoma oval, grey, middle of anteriorly and laterally with rod-shaped dark patterns, middle of posteriorly with inverted dark V-shaped patterns; venter with brown patterns and brown ring around spinnerets. Spinnerets base brown and tip white.

***Palp*** (Figs 12A, B, 17B). Patellar apophysis with wide base and narrowed tip, the narrowed tip as long as the wide base. Ventral retrolateral tibial apophysis almost straight, almost 1/2× longer than dorsal retrolateral tibial apophysis and as wide as dorsal retrolateral tibial apophysis. Cymbium 1.5× longer than wide. Cymbial furrow almost as long as cymbium. Bulb almost oval. A triangle process presents at the prolateral edge of tegulum. Medium of tegulum with oval membranous area. Length of stalk of conductor almost half of total conductor length. Embolic tooth terminal flat. Embolus slender and whip-shaped.

**Figure 18.**
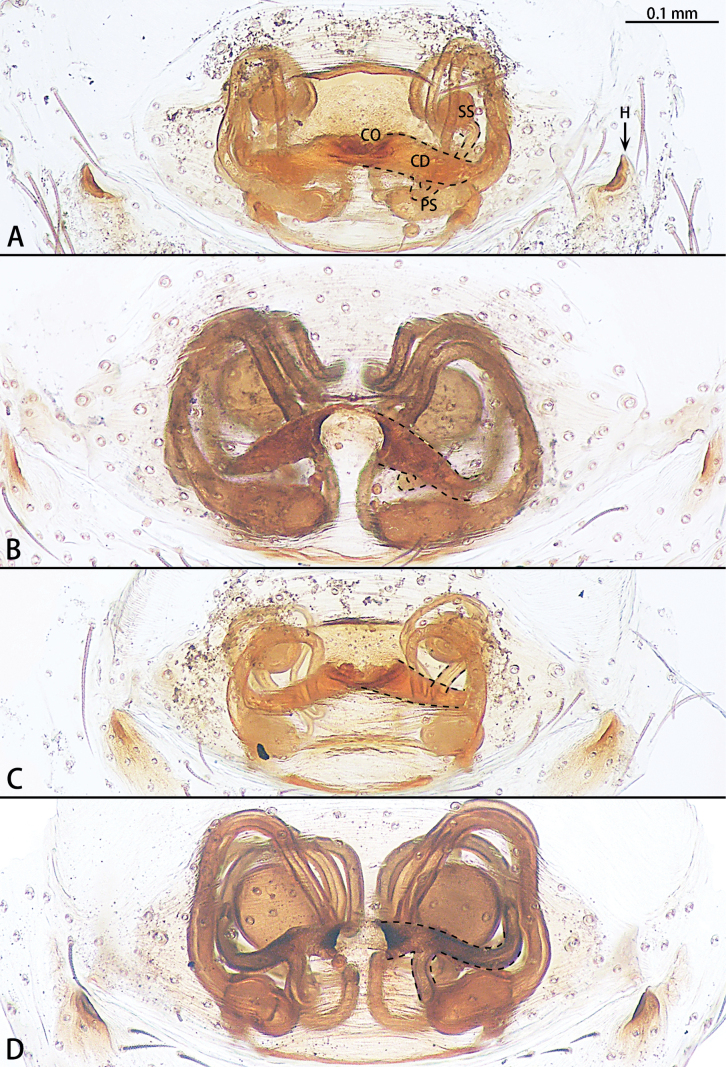
Epigynes of *Troglohnia* gen. nov., ventral view **A***Tr.dafang* sp. nov. **B***Tr.qiubei* sp. nov. **C***Tr.shidian* sp. nov. **D***Tr.wuding* sp. nov. Abbreviations: CD = copulatory duct, CO = copulatory opening, H = hood, PS = primary spermatheca, SS = secondary spermatheca.

**Figure 19.**
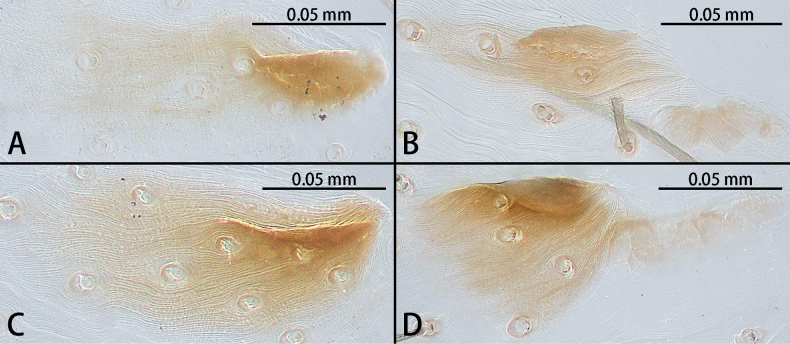
Epigynal hoods of *Troglohnia* gen. nov., ventral view **A***Tr.dafang* sp. nov. **B***Tr.qiubei* sp. nov. **C***Tr.shidian* sp. nov. **D***Tr.wuding* sp. nov.

**Figure 20.**
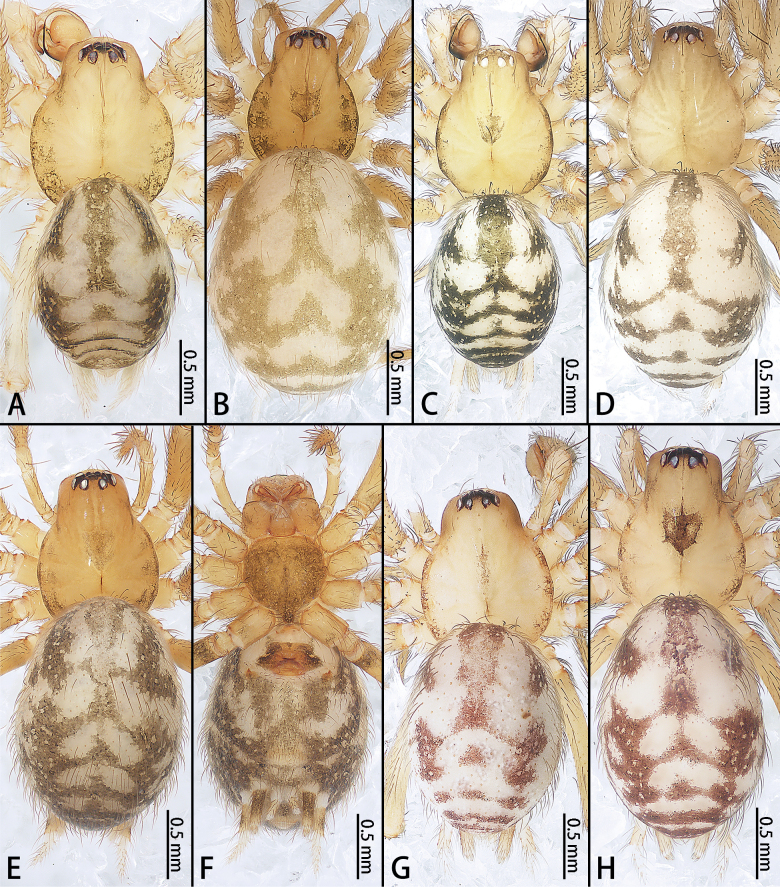
*Troglohnia* gen. nov., habitus, dorsal view (**A–E, G, H**) and ventral view (**F**) **A***Tr.dafang* sp. nov., holotype male **B** Same, paratype female **C***Tr.qiubei* sp. nov., holotype male **D** Same, paratype female **E***Tr.shidian* sp. nov., holotype female **F** Same **G***Tr.wuding* sp. nov., holotype male **H** same, paratype female.

**Female** (paratype IZCAS-Ar44682; Figs 13A, B, 18B, 19B, 20D). Total body length 3.39. Carapace 1.36 long, 1.04 wide; opisthosoma 2.03 long, 1.44 wide. Eight eyes with distinct black rims. Eye sizes and interdistances: AME 0.04, ALE 0.09, PME 0.08, PLE 0.09; AME–AME 0.03, AME–ALE 0.02, PME–PME 0.09, PME–PLE 0.04, ALE–PLE 0.02. MOA 0.20 long, front width 0.09, back width 0.23. Clypeus 0.17 high. Chelicerae with three promarginal and four retromarginal teeth. Leg measurements: I 4.51 (1.28, 0.46, 1.02, 0.99, 0.76); II 4.43 (1.28, 0.44, 0.98, 0.98, 0.75); III 4.25 (1.20, 0.44, 0.92, 1.03, 0.66); IV 5.37 (1.50, 0.45, 1.25, 1.32, 0.85). Leg supination: femora I–III d1; tibiae I v1, II v2, III p1 d2, r1, v3, IV p1 d2 r1 v2; metatarsi III p1 v2, IV p1 d2 r1 v1.

***Coloration*** (Fig. 20D). As in male but carapace without shield-shaped band.

***Epigyne*** (Figs 13A, B, 18B, 19B). Epigynal plate 1.45× wider than long. Hoods 3× depth than width. Copulatory openings arc-shaped, facing each other, circular. The width of thick copulatory ducts base 2× wider than the thinner part. The branched shorter copulatory ducts connected to the secondary spermathecae, the other connected to the primary spermathecae. Primary spermathecae bean-shaped, longer than the width of the secondary spermathecae. Fertilization ducts directed at 7:30 o’clock position from spermathecae.

##### Variation.

Males (*n* = 4): total body length 2.54–3.21, carapace 1.10–1.47 long, 0.80–1.22 wide, opisthosoma 1.44–1.74 long, 1.09–1.20 wide. Females (*n* = 4): total body length 3.03–3.62, carapace 1.28–1.44 long, 1.08–1.16 wide, opisthosoma 1.68–2.30 long, 1.19–1.48 wide.

##### Etymology.

The specific epithet refers to the type locality; noun in apposition.

##### Distribution.

Known only from the type locality (Fig. 30).

#### ﻿ Troglohnia shidian

Taxon classificationAnimaliaAraneaeHahniidae

Lin & Li sp. nov.

5865224C-6D13-5049-B0F6-343063A3B291

https://zoobank.org/5FBB3B13-DE99-4540-BD18-1EBBA8CCD693

Figs 14A, B , 18C , 19C , 20E, F , 30

##### Type material.

***Holotype***: ♀ (IZCAS-Ar44683), China, Yunnan: Baoshan City, Shidian County, Bailang Town, Xianren Cave, 24.6536°N, 99.2645°E, ca 1987 m, 29.VII.2010, C. Wang, Q. Zhao and Y. Lin leg. ***Paratype***: 1♀ (IZCAS-Ar44684), same data as holotype.

##### Diagnosis.

*Troglohniashidian* sp. nov. can be distinguished from those of *T.dafang* sp. nov. by the ratio of diameter of secondary spermathecae to length of branched shorter copulatory ducts almost 1:1 (Fig. 14B) [vs 1:2 (Fig. 11B)], primary spermathecae oval, separated by more than 2× diameters (Fig. 14B) [vs elongate bean-shaped, separated by less than one diameter (Fig. 11B)], diameter of primary spermathecae ~ 1.2× diameters of secondary spermathecae (Fig. 14B) [vs 2× (Fig. 11B)], distance between primary spermathecae and secondary spermathecae ~ 1× diameter of secondary spermathecae (Fig. 14B) [vs 2× (Fig. 11B)] and secondary spermathecae separated by ~ 3× diameters (Fig. 14B) [vs 4× (Fig. 11B)].

##### Description.

**Female** (holotype; Figs 14A, B, 18C, 19C, 20E, F). Total body length 3.75. Carapace 1.74 long, 1.33 wide; opisthosoma 2.01 long, 1.44 wide. AER straight and PER procurved slightly. Eye sizes and interdistances: AME 0.04, ALE 0.08, PME 0.07, PLE 0.09; AME–AME 0.03, AME–ALE 0.03, PME–PME 0.11, PME–PLE 0.06, ALE–PLE 0.02. MOA 0.17 long, front width 0.10, back width 0.24. Clypeus 0.15 high. Chelicerae with three promarginal and four retromarginal teeth. Leg measurements: I 3.72 (1.08, 0.40, 0.86, 0.78, 0.60); II 3.61 (1.07, 0.38, 0.83, 0.76, 0.57); III 3.39 (0.98, 0.37, 0.71, 0.78, 0.55); IV 4.23 (1.19, 0.40, 1.01, 1.00, 0.63). Leg supination: femora I p1, II–III d1; patellae III–III d1; tibiae I–II p1 d1, III p1 d2 r1 v2, IV p1 d1 r1 v2; metatarsi III p1 d1 v2, IV p1 d2 r1 v2.

***Coloration*** (Fig. 20E, F). Carapace yellow, with indistinct dark yellow radial grooves, middle region with shield-shaped brown band, margin with brown pattern. Fovea longitudinal, reddish-brown. Chelicerae, labium, gnathocoxae, and sternum yellow. Sternum with brown marking. Legs yellow. Opisthosoma oval, grey, middle of anteriorly and laterally with rod-shaped brown patterns, middle of posteriorly with inverted V-shaped brown patterns; venter with brown patterns and brown ring around spinnerets. Spinnerets base brown and tip white.

***Epigyne*** (Figs 14A, B, 18C, 19C). Epigynal plate 1.3× wider than long. Hoods 5× depth than width. The posterior edge of copulatory openings touching, slightly curved. The width of thick copulatory ducts base 3× wider than the thinner part. The branched shorter copulatory ducts connected to secondary spermathecae, the other connected to the primary spermathecae. Primary spermathecae oval, longer than the width of the secondary spermathecae. Fertilization ducts directed at 9:00 o’clock position from spermathecae.

##### Variation.

Second paratype female: total body length 3.47, carapace 1.35 long, 1.01 wide, opisthosoma 2.12 long, 1.42 wide.

##### Etymology.

The specific epithet refers to the type locality; noun in apposition.

##### Distribution.

Known only from the type locality (Fig. 30).

#### ﻿ Troglohnia wuding

Taxon classificationAnimaliaAraneaeHahniidae

Lin & Li sp. nov.

A7444512-F009-58A9-8B2D-31558EEB26F3

https://zoobank.org/B1F33F50-4AB0-4695-B217-38105B93C644

Figs 15A, B , 16A, B , 17C , 18D , 19D , 20G, H , 30

##### Type material.

***Holotype***: ♂ (IZCAS-Ar44685), China, Yunnan: Chuxiong City, Wuding County, Mao Street, Xianren Cave, 25.4655°N, 102.1740°E, ca 2066 m, 18.VI.2010, C. Wang, Q. Zhao and Y. Lin leg. ***Paratypes***: 10♀ (IZCAS-Ar44686–Ar44695), same data as holotype.

##### Diagnosis.

*Troglohniawuding* sp. nov. can be distinguished from *T.qiubei* sp. nov. by the male ventral retrolateral tibial apophysis strongly curved and wider than dorsal retrolateral tibial apophysis (Fig. 15B) [vs almost straight and as wide as dorsal retrolateral tibial apophysis (Fig. 12B)], embolic tooth terminal terminal finger-shaped (Fig. 15A) [vs flat (Fig. 12A)], process on anterior 3/5 of tegulum (arrowed in Fig. 15A) [vs on anterior 1/3 (arrowed in Fig. 12A)] and female can be distinguished by the diameter of secondary spermathecae almost as same as diameter of primary spermathecae (Fig. 16B) [vs less than diameter of primary spermathecae (Fig. 13B)], distance between primary spermathecae and secondary spermathecae less than half of diameter of secondary spermathecae (Fig. 16B) [vs more than half of diameter (Fig. 13B)], secondary spermathecae separated by ~ 1× diameter (Fig. 16B) [vs 1.5× (Fig. 13B)] and fertilization ducts pointing to 9:00 o’clock position (Fig. 16B) [vs 7:30 o’clock position (Fig. 13B)].

##### Description.

**Male** (holotype; Figs 15A, B, 17C, 20G). Total body length 2.88. Carapace 1.27 long, 1.02 wide; opisthosoma 1.61 long, 1.19 wide. AER straight and PER procurved in dorsal view. Eye sizes and interdistances: AME 0.05, ALE 0.07, PME 0.05, PLE 0.06; AME–AME 0.01, AME–ALE 0.03, PME–PME 0.08, PME–PLE 0.04, ALE–PLE 0.01. MOA 0.18 long, front width 0.12, back width 0.17. Clypeus 0.16 high. Chelicerae with two promarginal and three retromarginal teeth. Leg measurements: I 4.36 (1.27, 0.43, 1.00, 0.93, 0.73); II 4.19 (1.25, 0.41, 0.94, 0.89, 0.70); III 3.81 (1.11, 0.40, 0.81, 0.86, 0.63); IV 4.73 (1.30, 0.41, 1.14, 1.12, 0.76). Leg spination: femora I–III d1; tibiae I p1 d1, II d2, III p1 d2 r1 v2, IV r1 v2; metatarsi III–IV p1 r1 v2.

***Coloration*** (Fig. 20G). Carapace yellowish, middle region with elongated brown band, margin with brown pattern. Fovea longitudinal, reddish-brown. Chelicerae, labium, gnathocoxae, and sternum yellow. Sternum with brown marginal marking. Legs yellow. Dorsal opisthosoma oval, grey, middle of anteriorly and laterally with rod-shaped brown patterns, middle of posteriorly with dotted brown patterns. Ventral opisthosoma grey, anterior and middle part with brown patterns. Spinnerets surrounded by brown rings. Spinnerets base brown and tip white.

***Palp*** (Figs 15A, B, 17C). Patellar apophysis with wide base and narrowed tip, the narrowed tip as long as the wide base. Ventral retrolateral tibial apophysis curved, almost 1/2× longer than dorsal retrolateral tibial apophysis and 2× wider than the dorsal retrolateral tibial apophysis. Cymbium 1.5× longer than wide. Cymbial furrow almost as long as cymbium. Bulb almost oval. A triangle process presents at the prolateral edge of tegulum. Medium of tegulum with oval membranous area. Length of stalk of conductor almost half of total conductor length. Embolic tooth terminal blunt. Embolus slender and whip-shaped.

**Female** (paratype IZCAS-Ar44695; Figs 16A, B, 18D, 19D, 20H). Total body length 2.26. Carapace 0.93 long, 0.72 wide; opisthosoma 1.33 long, 0.90 wide. Eye sizes and interdistances: AME 0.05, ALE 0.07, PME 0.07, PLE 0.08; AME–AME 0.02, AME–ALE 0.03, PME–PME 0.08, PME–PLE 0.04, ALE–PLE 0.02. MOA 0.18 long, front width 0.07, back width 0.19. Clypeus 0.13 high. Chelicerae with three promarginal and four retromarginal teeth. Leg measurements: I 3.55 (1.03, 0.37, 0.81, 0.77, 0.57); II 3.45 (1.02, 0.36, 0.76, 0.75, 0.56); III 3.15 (0.84, 0.35, 0.70, 0.74, 0.52); IV 4.03 (1.09, 0.39, 0.95, 0.99, 0.61). Leg spination: femora I–III d1; patellae I–IV d1; tibiae I d1, II p1 d2, III–IV p1 d2 r1 v2; metatarsi III–IV p1 r1 v2.

***Coloration*** (Fig. 20H). As in male but carapace with shield-shaped brown band at middle.

***Epigyne*** (Figs 16A, B, 18D, 19D). Epigynal plate 1.35× wider than long. Hoods 3× depth than width. Copulatory openings arc-shaped, facing each other, circular. The width of thick copulatory ducts base 2× wider than the thinner part. The branched shorter copulatory ducts connected to the secondary spermathecae, the other connected to the primary spermathecae. Primary spermathecae bean-shaped, almost as wide as the secondary spermathecae. Fertilization ducts directed at 9:00 o’clock position from spermathecae.

##### Variation.

Females (*n* = 9): total body length 3.06–3.86, carapace 1.26–1.58 long, 1.00–1.23 wide, opisthosoma 1.80–2.33 long, 1.29–1.74 wide.

##### Etymology.

The specific epithet refers to the type locality; noun in apposition.

##### Distribution.

Known only from the type locality (Fig. 30).

#### ﻿Genus Typhlohnia

Taxon classificationAnimaliaAraneaeHahniidae

Lin & Li gen. nov.

B9FA4BF9-5735-5C1E-8F12-4DFC1154D8D3

https://zoobank.org/4FDC2FD9-21F4-457C-A0A9-3F2BD5B6AA06

##### Type species.

*Typhlohniarongshui* sp. nov. from Guangxi, China.

##### Diagnosis.

**Figure 21.**
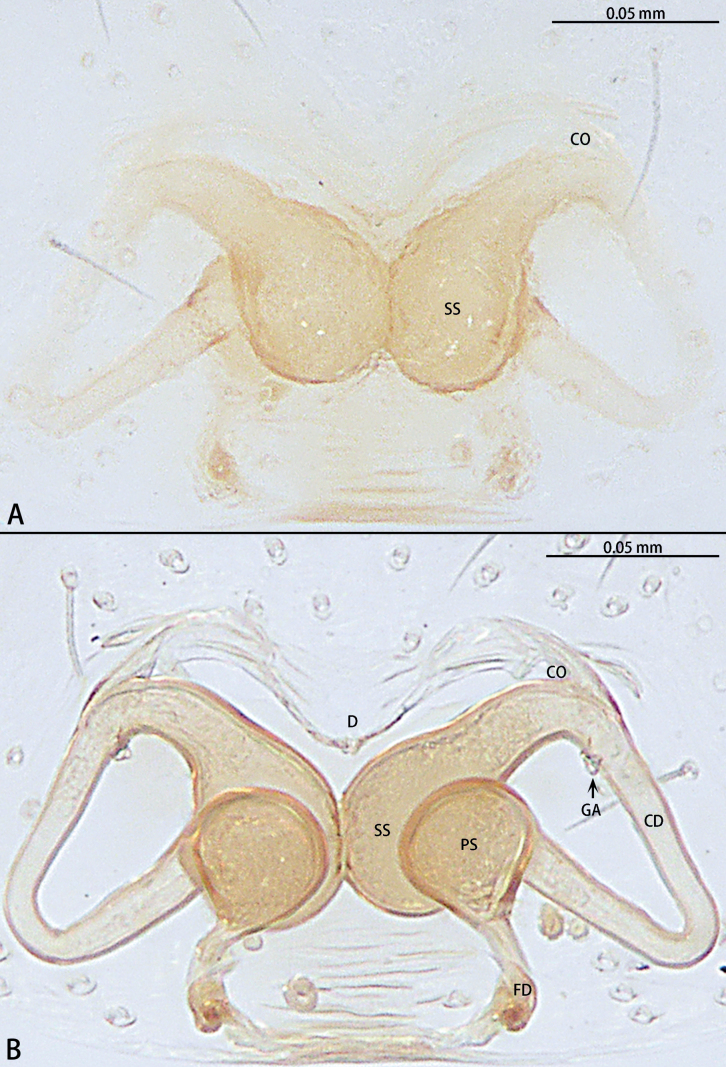
*Typhlohniabanlaksao* sp. nov., holotype female **A** epigyne, ventral view **B** vulva, dorsal view. Abbreviations: CD = copulatory duct, CO = copulatory opening, D = depression, FD = fertilization duct, GA = glandular appendage, PS = primary spermatheca, SS = secondary spermatheca.

**Figure 22.**
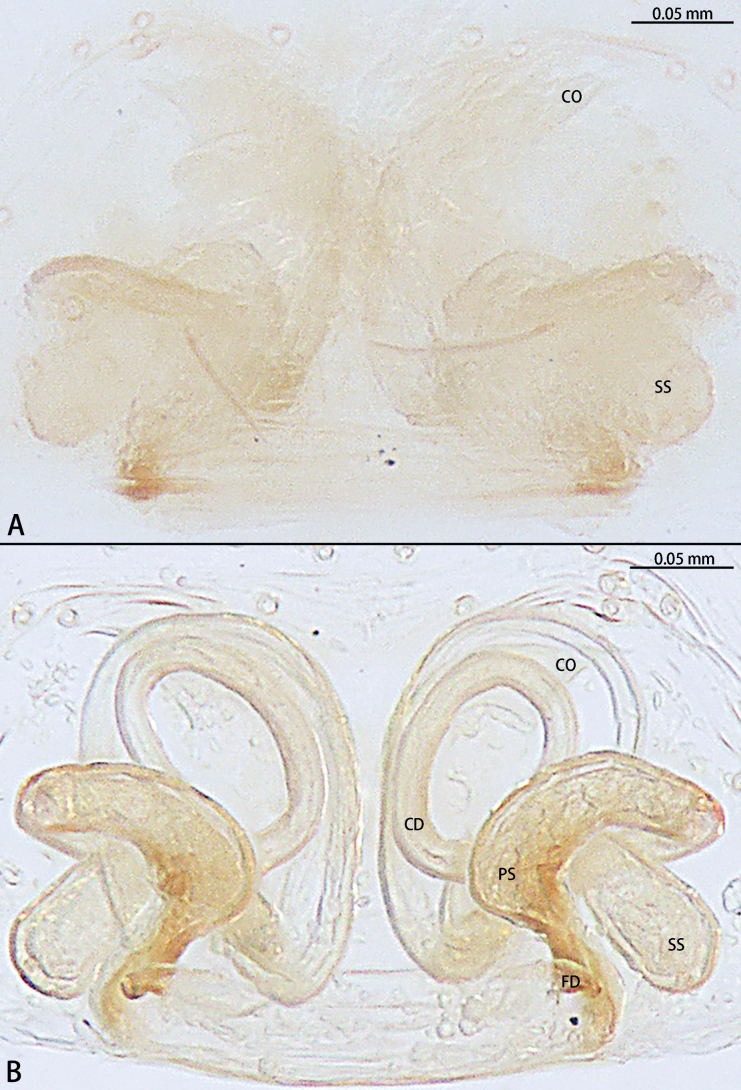
*Typhlohniakaiyang* sp. nov., holotype female **A** epigyne, ventral view **B** vulva, dorsal view. Abbreviations: CD = copulatory duct, CO = copulatory opening, FD = fertilization duct, PS = primary spermatheca, SS = secondary spermatheca.

**Figure 23.**
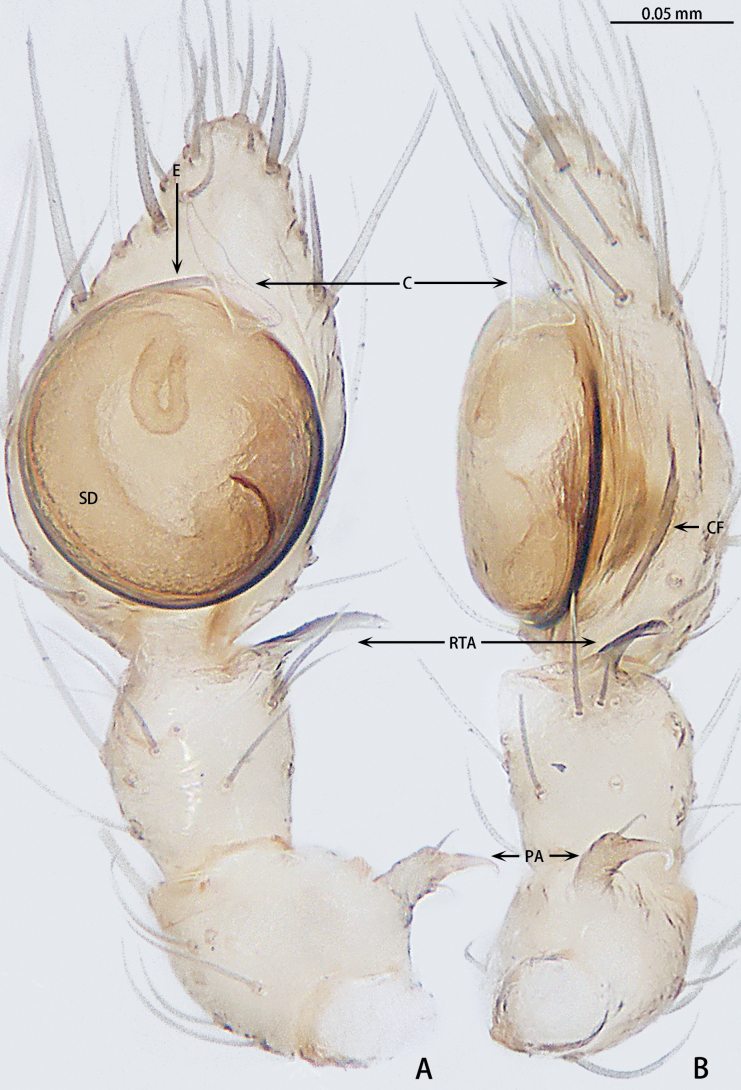
*Typhlohniarongshui* sp. nov., holotype male **A** ventral view **B** retrolateral view. Abbreviations: C = conductor, CF = cymbial furrow, E = embolus, PA = patellar apophysis, RTA = retrolateral tibial apophysis, SD = sperm duct.

**Figure 24.**
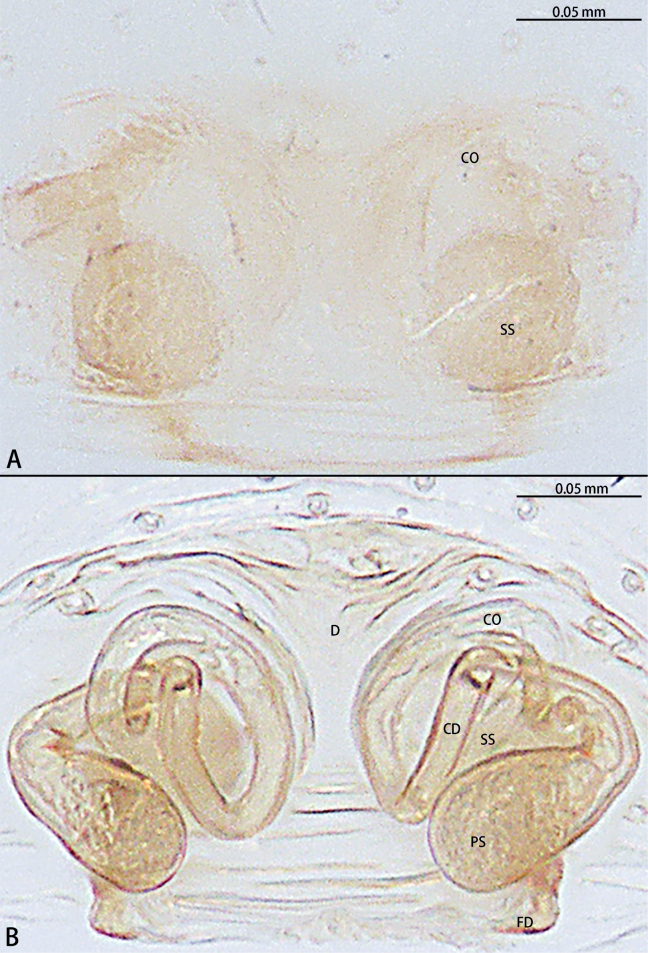
*Typhlohniarongshui* sp. nov., paratype female **A** epigyne, ventral view **B** vulva, dorsal view. Abbreviations: CD = copulatory duct, CO = copulatory opening, D = depression, FD = fertilization duct, PS = primary spermatheca, SS = secondary spermatheca.

**Figure 25.**
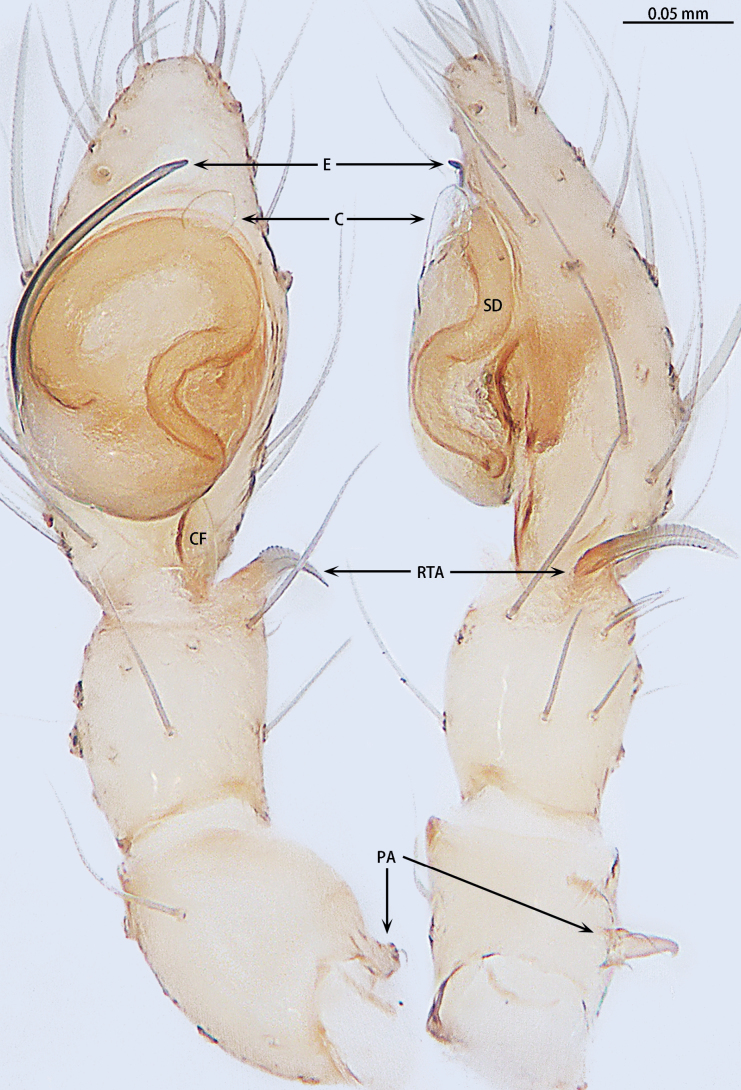
*Typhlohniasondoong* sp. nov., holotype male **A** ventral view **B** retrolateral view. Abbreviations: C = conductor, CF = cymbial furrow, E = embolus, PA = patellar apophysis, RTA = retrolateral tibial apophysis, SD = sperm duct.

**Figure 26.**
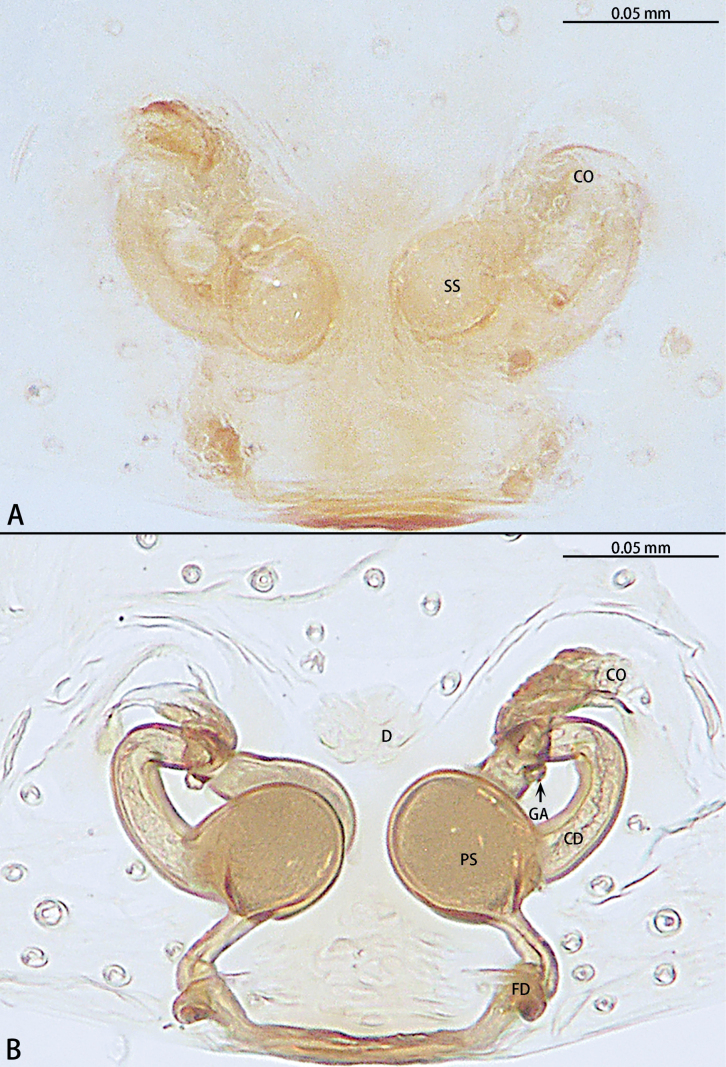
*Typhlohniasondoong* sp. nov., paratype female **A** epigyne, ventral view **B** vulva, dorsal view. Abbreviations: CD = copulatory duct, CO = copulatory opening, D = depression, FD = fertilization duct, GA = glandular appendage, PS = primary spermatheca, SS = secondary spermatheca.

**Figure 27.**
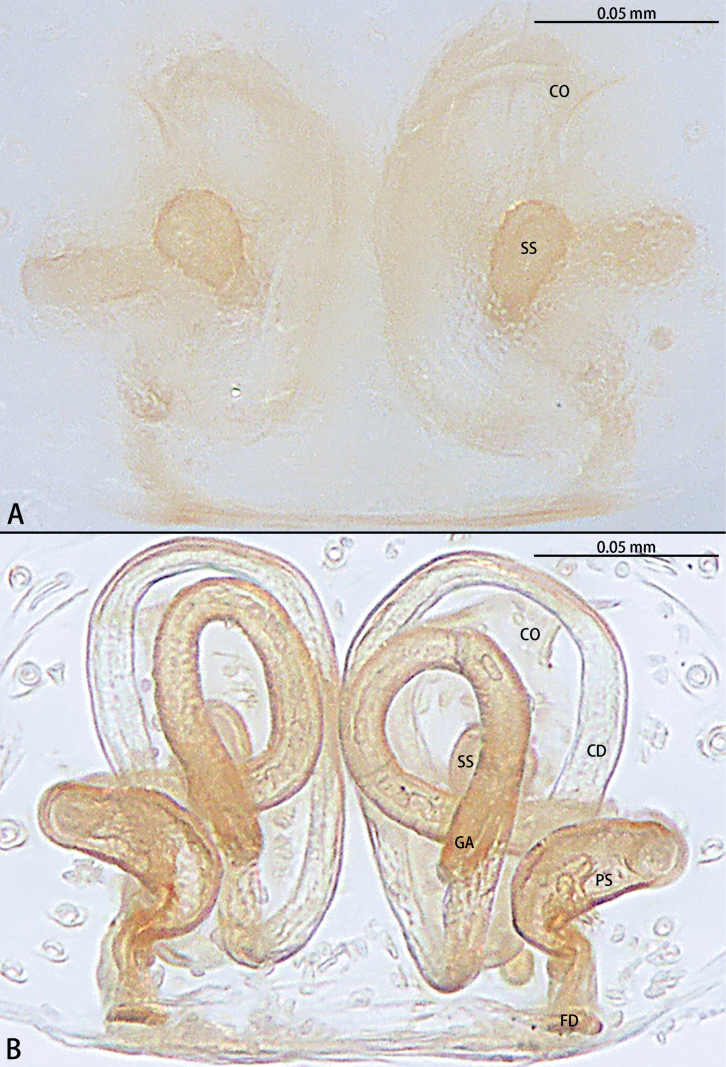
*Typhlohniasuiyang* sp. nov., holotype female **A** epigyne, ventral view **B** vulva, dorsal view. Abbreviations: CD = copulatory duct, CO = copulatory opening, FD = fertilization duct, GA = glandular appendage, PS = primary spermatheca, SS = secondary spermatheca.

**Figure 28.**
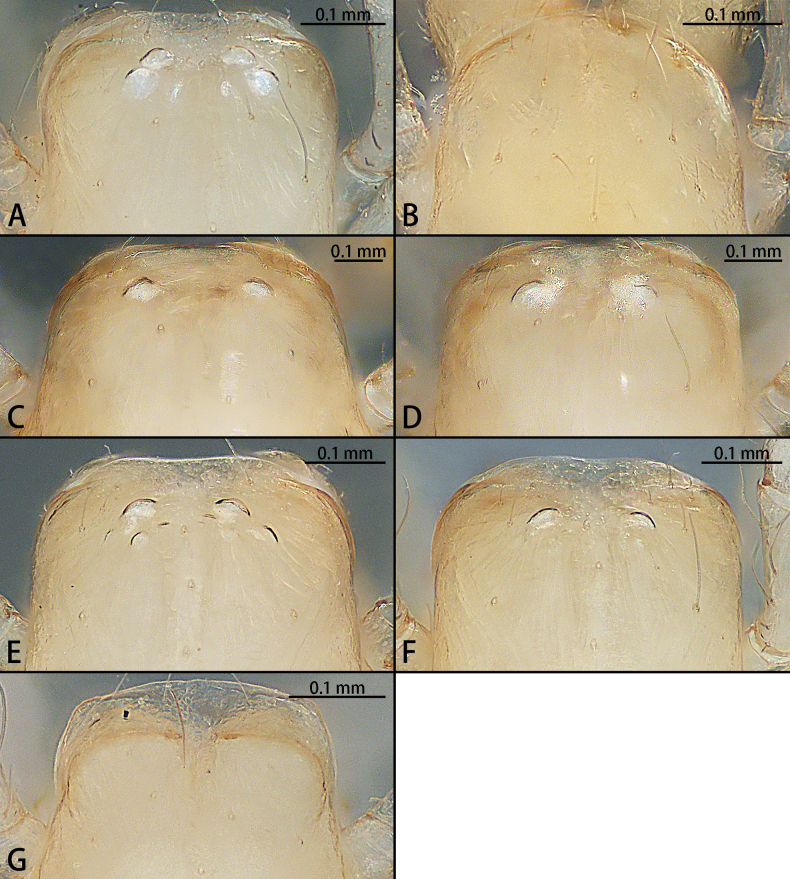
Cephalic regions of *Typhlohnia* gen. nov., dorsal view **A***Ty.banlaksao* sp. nov., holotype female **B***Ty.kaiyang* sp. nov., holotype female **C***Ty.rongshui* sp. nov., holotype male **D** Same, paratype female **E***Ty.sondoong* sp. nov., holotype male **F** same, paratype female **G***Ty.suiyang* sp. nov., holotype female.

**Figure 29.**
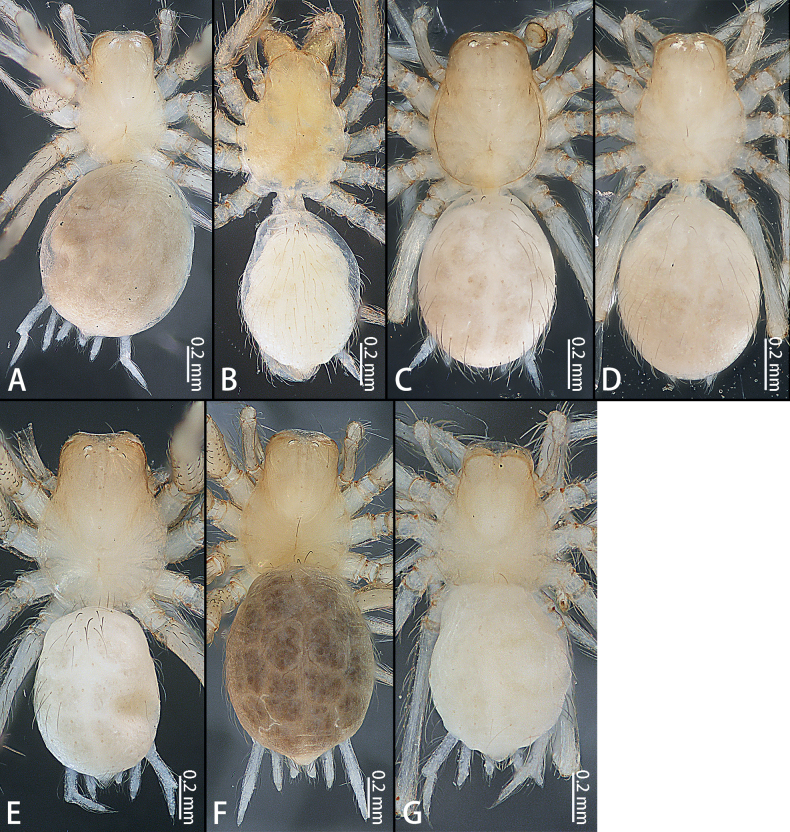
*Typhlohnia* gen. nov., habitus, dorsal view **A***Ty.banlaksao* sp. nov., holotype female **B***Ty.kaiyang* sp. nov., holotype female **C***Ty.rongshui* sp. nov., holotype male **D** Same, paratype female **E***Ty.sondoong* sp. nov., holotype male **F** same, paratype female **G***Ty.suiyang* sp. nov., holotype female.

*Typhlohnia* gen. nov. can be distinguished from *Asiohahnia* Ovtchinnikov, 1992 by the eyes retrograde (Fig. 28A–G) [vs eyes normal (see Ovtchinnikov 1992: fig. 2.3)], body pale yellow to white (Fig. 29A–G) [vs body with black patterns (see Ovtchinnikov 1992: fig. 2.4)], the length of cymbium almost 3–6× of the length of cymbial furrow (Figs 23B, 25B) [vs 2× (see Ovtchinnikov 1992: figs 2.2, 3.2)], sperm duct with U-shaped curve (Figs 3D, 23A, 25A) [vs without curved (see Ovtchinnikov 1992: figs 2.2, 2.6, 3.2)], copulatory openings anteriorly (Figs 21A, 22A, 24A, 26A, 27A) [vs posteriorly (see Ovtchinnikov 1992: figs 3.3, 3.5, 3.7)] and epigyne with two pairs of spermathecae (Figs 21B, 22B, 24B, 26B, 27B) [vs one pair (see Ovtchinnikov 1992: figs 3.4, 3.6, 3.8)].

##### Description.

**Male.** Total length 1.38–1.70 (*n* = 4). Carapace pale white to yellowish, without any pattern. 0–6 eyes, white, most species with two eyes. Fovea longitudinal, unobvious. Clypeus pale yellow, covered with several setae. Chelicerae pale yellow, with two or three promarginal and two or three retromarginal teeth, stridulatory files absent. Endites, labium pale yellow, covered with few black setae. Sternum brown, without markings. Legs pale yellow. Opisthosoma oval, pale white to brown. Spinnerets white, straight in posterior view. Tracheal spiracle long and transverse, distance of spiracle to epigastric furrow as long as to spinnerets.

Palpal femur almost 3× longer than patella, spineless. Patella almost as long as tibia, with hook-shaped apophysis. Retrolateral tibial apophysis curved with serrations. Cymbium oval, almost 2× longer than wide, cymbial furrow almost 1/3–1/6× longer than cymbium. Bulb globular to oval. Sperm duct with U-shaped curve. Embolus whip-shaped, curving clockwise along tegular margin.

**Female.** Total length 1.30–2.07 (*n* = 13). Somatic characters as in male.

Epigynal plate wider than long, with a depression anteriorly. Copulatory openings located anteriorly, arc-shaped. Copulatory ducts long, in the *rongshui* group strongly convoluted, but in the *sondoong* group simple. The short duct connected to secondary spermathecae, the other connected to primary spermathecae. Primary spermathecae oval to bean-shaped, secondary spermathecae oval to globular. Fertilization ducts laminar, sickle-shaped.

##### Etymology.

The new generic name is a combination of *Typhlo*- (refers to the degenerated eyes) and *Hahnia*. The gender is feminine.

##### Species groups.

Two species groups: the *rongshui* group and the *sondoong* group. These groups can be distinguished by the males embolus originating at 3:00 o’clock position (the *rongshui* group) or 7:30 o’clock position (the *sondoong* group), length of embolus almost 3/4 perimeter of bulb (the *rongshui* group) or half perimeter of bulb (the *sondoong* group) and females have convoluted copulatory ducts (the *rongshui* group) or simple copulatory ducts (the *sondoong* group).

##### Composition.

This new genus includes five species: The *rongshui* group: *Typhlohniakaiyang* sp. nov. (♀), *T.rongshui* sp. nov. (♂♀) and *T.suiyang* sp. nov. (♀) and the *sondoong* group: *T.banlaksao* sp. nov. (♀) and *T.sondoong* sp. nov. (♂♀).

##### Distribution.

**Figure 30.**
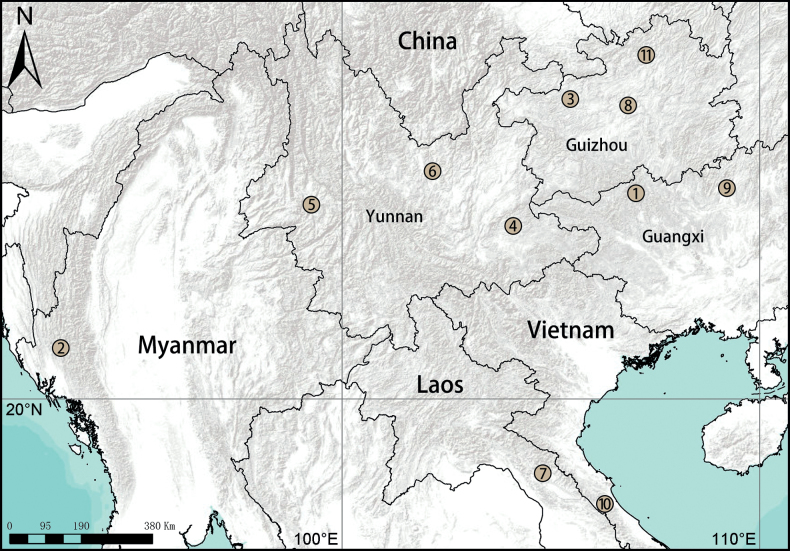
Distribution records of new Hahniidae species in South-east Asia **1***Gobliniatiane* sp. nov. **2***Myahniakanpetlet* sp. nov. **3***Troglohniadafang* sp. nov. **4***Tr.qiubei* sp. nov. **5***Tr.shidian* sp. nov. **6***Tr.wuding* sp. nov. **7***Typhlohniabanlaksao* sp. nov. **8***Ty.kaiyang* sp. nov. **9***Ty.rongshui* sp. nov. **10***Ty.sondoong* sp. nov. **11***Ty.suiyang* sp. nov.

Laos (Bolikhamxay), Vietnam (Quang Binh) and China (Guizhou, Guangxi) (Fig. 30).

#### ﻿ Typhlohnia banlaksao

Taxon classificationAnimaliaAraneaeHahniidae

Lin & Li sp. nov.

857B5D75-CD3F-5D27-8B71-8868DDECFEF0

https://zoobank.org/977A9612-279D-4DBF-B8D3-26D5CB5F7AC7

Figs 21A, B , 28A , 29A , 30

##### Type material.

***Holotype***: ♀ (IZCAS-Ar44696), Laos, Bolikhamxay: Khamkeut Dist., 17.11 km west of Ban Laksao Town, Tham Mankone, Dragon Cave, 18.2216°N, 104.8127°E, ca 495 m, 27.XI.2012, Z. Yao leg.

##### Diagnosis.

The female of *Typhlohniabanlaksao* sp. nov. can be distinguished from *T.sondoong* sp. nov. by the length of copulatory ducts 4× longer than diameter of primary spermathecae (Fig. 21B) [vs 1.5× (Fig. 26B)], copulatory ducts strongly curved to almost 60° angle (Fig. 21B) [vs 80° (Fig. 26B)] and secondary spermathecae larger than primary spermathecae (Fig. 21B) [vs as wide as primary spermathecae (Fig. 26B)].

##### Description.

**Female** (holotype; Figs 21A, B, 28A, 29A). Total body length 1.73. Carapace 0.74 long, 0.49 wide; opisthosoma 0.99 long, 0.77 wide. Eye sizes and interdistances: ALE 0.02, PME 0.01, PLE 0.03; PME–PME 0.05, PME–PLE 0.02, ALE–PLE 0.02. Clypeus 0.09 high. Chelicerae with three promarginal and two retromarginal teeth. Leg measurements: I 3.03 (0.84, 0.26, 0.71, 0.67, 0.55); II 2.80 (0.79, 0.26, 0.62, 0.64, 0.49); III 2.58 (0.70, 0.22, 0.57, 0.62, 0.47); IV 3.09 (0.91, 0.26, 0.81, 0.84, 0.57). Leg spination: femur I p1; patellae I–IV d1; tibiae I–II p1 d2, III–IV r1 d1.

***Coloration*** (Figs 28A, 29A). Carapace pale yellow, with a few long brown hairs. Fovea longitudinal, reddish-brown. Six eyes, white. Chelicerae, labium, and gnathocoxae pale yellow, with long brown hairs; sternum yellowish. Legs white with some spines. Opisthosoma oval, grey. Spinnerets white.

***Epigyne*** (Fig. 21A, B). Epigynal plate 1.55× wider than long. Depression obvious, ends with copulatory openings. Copulatory ducts long, almost 4× longer than width of primary spermathecae, strongly curved to almost 60° angle, base bifurcate. The short one connected to secondary spermathecae, the other connected to primary spermathecae. Secondary spermathecae oval, 1.5× wider than primary spermathecae. Fertilization ducts directed at 11:00 o’clock position from spermathecae.

##### Etymology.

The specific epithet refers to the type locality; noun in apposition.

##### Distribution.

Known only from the type locality (Fig. 30).

#### ﻿ Typhlohnia kaiyang

Taxon classificationAnimaliaAraneaeHahniidae

Lin & Li sp. nov.

99E48643-5169-5213-91E6-099B9A5A9889

https://zoobank.org/865144DC-09CE-4C98-A0F7-C4675AE7BCC3

Figs 22A, B , 28B , 29B , 30

##### Type material.

***Holotype***: ♀ (IZCAS-Ar44697), China, Guizhou: Guiyang City, Kaiyang County, Shuangliu Town, Dashan Villiage, Qiaotou Cave, 27.0316°N, 106.8571°E, ca 1380 m, 11.V.2006, Y. Lin and Z. Yang leg.

##### Diagnosis.

The female of *Typhlohniakaiyang* sp. nov. can be distinguished from all other species in the *rongshui* group by the secondary spermathecae at posterior of primary spermathecae (Fig. 22B).

##### Description.

**Female** (holotype; Figs 22A, B, 28B, 29B). Total body length 1.53. Carapace 0.66 long, 0.48 wide; opisthosoma 0.87 long, 0.50 wide. Chelicerae with two promarginal and three retromarginal teeth. Leg measurements: I 2.03 (0.58, 0.22, 0.44, 0.40, 0.38); II 1.97 (0.57, 0.21, 0.41, 0.41, 0.37); III 1.80 (0.50, 0.17, 0.39, 0.39, 0.35); IV 2.35 (0.65, 0.22, 0.51, 0.54, 0.43). Leg spination: patellae III–IV d1.

***Coloration*** (Figs 28B, 29B). Carapace pale yellow, with a few long brown hairs. Fovea unobvious. Eyes absent. Chelicerae, labium, and gnathocoxae pale yellow, with long brown hairs. Sternum yellowish. Legs white. Opisthosoma oval, white. Spinnerets white.

***Epigyne*** (Fig. 22A, B). Epigynal plate 1.3× wider than long. Depression unobvious. Copulatory openings arc-shaped. Copulatory ducts long and strongly convoluted, with two turns, medium bifurcate. The short one connected to secondary spermathecae, the other connected to primary spermathecae. Secondary spermathecae oval, as wide as kidney-shaped primary spermathecae. Fertilization ducts directed at 9:00 o’clock position from spermathecae.

##### Etymology.

The specific epithet refers to the type locality; noun in apposition.

##### Distribution.

Known only from the type locality (Fig. 30).

#### ﻿ Typhlohnia rongshui

Taxon classificationAnimaliaAraneaeHahniidae

Lin & Li sp. nov.

044C9042-CD3B-5D58-9750-CA7831A488D0

https://zoobank.org/E78901D0-F6DA-4033-9D07-5CE389BE5FC3

Figs 1D , 3D , 23A, B , 24A, B , 28C, D , 29C, D , 30

##### Type material.

***Holotype***: ♂ (IZCAS-Ar44698), China, Guangxi: Guilin City, Rongshui County, Taoyuan Cave, 25.0579°N, 109.2246°E, ca 131 m, 23.VII.2009, C. Wang leg. ***Paratypes***: 3♂ 4♀ (IZCAS-Ar44699–Ar44705), same data as holotype.

##### Diagnosis.

The male of *Typhlohniarongshui* sp. nov. can be distinguished from *T.sondoong* sp. nov. by the patella with apophysis retrolaterally (Fig. 23B) [vs retrodorsally (Fig. 25B)], retrolateral tibial apophysis point retrolaterally (Fig. 23B) [vs point dorsally (Fig. 25B)], conductor slender and triangle-shaped (Fig. 23A) [vs oval (Fig. 25A)], medium of sperm duct U-shaped (Fig. 23A) [vs upturned U-shaped (Fig. 25A)], embolus originating at 3:00 o’clock position (Fig. 23A) [vs 7:30 o’clock position (Fig. 25A)] and length of embolus almost 3/4 perimeter of bulb (Fig. 23A) [vs half perimeter of bulb (Fig. 25A)]. Females can be distinguished from *T.suiyang* sp. nov. by the unobvious glandular appendages (Fig. 24B) [vs obvious (Fig. 27B)], ratio of length to width of epigynal plate ~ 2:3 (Fig. 24A) [vs 1:1 (Fig. 27A)] and ratio of width between primary spermathecae to secondary spermathecae ~ 1:1 (Fig. 24B) [vs 2:1 (Fig. 27B)].

##### Description.

**Male** (holotype; Figs 23A, B, 28C, 29C). Total body length 1.39. Carapace 0.63 long, 0.48 wide; opisthosoma 0.76 long, 0.56 wide. Eye size and interdistance: PLE 0.03; PLE–PLE 0.11. Clypeus 0.07 high. Chelicerae with two promarginal and three retromarginal teeth. Leg measurements: I 1.98 (0.60, 0.23, 0.45, 0.37, 0.33); II 1.85 (0.56, 0.21, 0.40, 0.35, 0.33); III 1.68 (0.51, 0.18, 0.34, 0.34, 0.31); IV 2.10 (0.61, 0.21, 0.46, 0.45, 0.37). Leg spination: femur I p1; patellae III–IV d1; tibiae III–IV p1 d1; metatarsi III p1 d2, IV d2.

***Coloration*** (Figs 28C, 29C). Carapace pale yellow, with a few long brown hairs. Fovea longitudinal, reddish-brown. Two eyes, white. Chelicerae, labium, and gnathocoxae pale yellow, with long brown hairs; sternum pale yellowish. Legs white. Opisthosoma oval, pale yellow. Spinnerets white.

***Palp*** (Fig. 23A, B). Patella with apophysis retrolaterally, tip hook-shaped. Tibia with black, serrated retrolateral apophysis, little curved, point retrolaterally. Cymbium 1.5× longer than wide, 3× longer than cymbial furrow. Cymbial furrow shallowed. Bulb globular. Conductor sickle-shaped, almost half length of bulb. Middle of sperm duct bent in U-shape. Embolus slender and whip-shaped, almost 3/4 perimeter of bulb. Base of embolus arising at 3:00 o’clock position.

**Female** (paratype IZCAS-Ar44705; Figs 24A, B, 28D, 29D). Total body length 1.34. Carapace 0.59 long, 0.47 wide; opisthosoma 0.75 long, 0.48 wide. Eye size and interdistance: PLE 0.03; PLE–PLE 0.09. Clypeus 0.06 high. Chelicerae with two promarginal and three retromarginal teeth. Leg measurements: I 1.84 (0.56, 0.20, 0.41, 0.35, 0.32); II 1.76 (0.54, 0.19, 0.38, 0.34, 0.31); III 1.64 (0.49, 0.17, 0.35, 0.33, 0.30); IV 2.07 (0.59, 0.20, 0.46, 0.45, 0.37). Leg spination: patellae III–IV d1; tibiae II–IV r1; metatarsi III–IV p1 d2 r1.

***Coloration*** (Figs 28D, 29D). As in male.

***Epigyne*** (Fig. 24A, B). Epigynal plate 1.5× wider than long. Depression obvious, ends with copulatory openings. Copulatory openings arc-shaped. Copulatory ducts long and strongly convoluted with one turn, medium bifurcate. The short one connected to the secondary spermathecae, the other connected to the primary spermathecae. Secondary spermathecae globular, as wide as bean-shaped primary spermathecae. Fertilization ducts directed at 9:00 o’clock position from spermathecae.

##### Variation.

Males (*n* = 2): total body length 1.38–1.46, carapace 0.68–0.71 long, 0.51–0.59 wide, opisthosoma 0.70–0.75 long, 0.55–0.57 wide. Females (*n* = 3): total body length 1.30–1.40, carapace 0.55–0.61 long, 0.44–0.46 wide, opisthosoma 0.73–0.81 long, 0.50–0.57 wide.

##### Etymology.

The specific epithet refers to the type locality; noun in apposition.

##### Distribution.

Known only from the type locality (Fig. 30).

#### ﻿ Typhlohnia sondoong

Taxon classificationAnimaliaAraneaeHahniidae

Lin & Li sp. nov.

245278E0-51EB-5166-8C98-1ED675F21FF6

https://zoobank.org/B1DE5788-66C5-4408-9737-9993FB969A70

Figs 25A, B , 26A, B , 28E, F , 29E, F , 30

##### Type material.

***Holotype***: ♂ (IZCAS-Ar44706), Vietnam, Quang Binh: Phong Nha-Ke Bang National Park, Son Doong Cave, 17.4936°N, 106.2942°E, ca 143 m, 25.V.2016, Q. Zhao and Z. Chen leg. ***Paratypes***: 6♀ (IZCAS-Ar44707–Ar44712), same data as holotype.

##### Diagnosis.

For males see diagnosis of *Typhlohniarongshui* sp. nov. and for females see diagnosis of *T.banlaksao* sp. nov.

##### Description.

**Male** (holotype; Figs 25A, B, 28E, 29E). Total body length 1.70. Carapace 0.83 long, 0.64 wide; opisthosoma 0.87 long, 0.62 wide. Eye sizes and interdistance: ALE 0.03, PLE 0.02; ALE–PLE 0.03. Clypeus 0.12 high. Chelicerae with three promarginal and two retromarginal teeth. Leg measurements: I 2.84 (0.80, 0.26, 0.68, 0.62, 0.48); II 2.71 (0.77, 0.25, 0.62, 0.61, 0.46); III 2.50 (0.69, 0.24, 0.56, 0.58, 0.43); IV 3.11 (0.85, 0.24, 0.76, 0.74, 0.52). Leg spination: femora I p1 v4, II–IV v4; patellae II–IV d1; tibiae I p1, II p1 d1, III–IV p1 r1; metatarsi III–IV p1 r1 v1.

***Coloration*** (Figs 28E, 29E). Carapace pale yellow, with a few long brown hairs. Fovea longitudinal, reddish-brown. Four eyes, white. Chelicerae, labium, and gnathocoxae pale yellow, with long brown hairs; sternum yellowish. Legs yellow. Opisthosoma oval, pale yellow. Spinnerets white.

***Palp*** (Fig. 25A, B). Patella with apophysis retrodorsally, tip hook-shaped. Tibia with black, serrated retrolateral apophysis, little curved, point dorsally. Cymbium 2× longer than wide, 6× longer than cymbial furrow. Cymbial furrow shallowed, originated on the inside of cymbium. Bulb oval. Conductor oval, almost as wide as middle of sperm duct. Middle of sperm duct bent in upturned U-shape. Embolus slender and whip-shaped, almost half the perimeter of bulb. Base of embolus arising at 7:30 o’clock position.

**Female** (paratype IZCAS-Ar44712; Figs 26A, B, 28F, 29F). Total body length 1.73. Carapace 0.74 long, 0.55 wide; opisthosoma 0.99 long, 0.76 wide. Eye sizes and interdistance: ALE 0.03, PLE 0.02; ALE–PLE 0.03. Clypeus 0.10 high. Chelicerae with three promarginal and two retromarginal teeth. Leg measurements: I 2.54 (0.74, 0.24, 0.58, 0.54, 0.44); II 2.44 (0.72, 0.23, 0.53, 0.53, 0.43); III 2.30 (0.64, 0.22, 0.52, 0.52, 0.40); IV 2.89 (0.80, 0.25, 0.71, 0.66, 0.47). Leg spination: femora I p1 v4, II–IV v4; tibiae III–IV p1; metatarsi III–IV p1 r1 v1.

***Coloration*** (Figs 28F, 29F). As in male.

***Epigyne*** (Fig. 26A, B). Epigynal plate 1.1× wider than long. Depression obvious, ends with copulatory openings. Copulatory ducts long, almost 1.5× longer than width of primary spermathecae, strongly curved to almost 80° angle, base bifurcate. The short one connected to secondary spermathecae, the other connected to primary spermathecae. Secondary spermathecae oval, as wide as primary spermathecae. Fertilization ducts directed at 9:00 o’clock position from spermathecae.

##### Variation.

Females (*n* = 5): total body length 1.50–2.07, carapace 0.69–0.80 long, 0.51–0.59 wide, opisthosoma 0.75–1.27 long, 0.61–1.02 wide.

##### Etymology.

The specific epithet refers to the type locality; noun in apposition.

##### Distribution.

Known only from the type locality (Fig. 30).

#### ﻿ Typhlohnia suiyang

Taxon classificationAnimaliaAraneaeHahniidae

Lin & Li sp. nov.

99C24189-BE0A-5072-8E2B-625D6B67229D

https://zoobank.org/0855F4D9-0B3E-407C-85A7-92782883645D

Figs 27A, B , 28G , 29G , 30

##### Type material.

***Holotype***: ♀ (IZCAS-Ar44713), China, Guizhou: Suiyang County, Wenquan Town, Guihua Villiage, Mahuang Cave, 28.2437°N,107.2891°E, ca 730 m, 13.V.2007, Y. Lin and J. Liu leg.

##### Diagnosis.

See diagnosis of *Typhlohniarongshui* sp. nov.

##### Description.

**Female** (holotype; Figs 27A, B, 28G, 29G). Total body length 1.31. Carapace 0.61 long, 0.49 wide; opisthosoma 0.70 long, 0.57 wide. Chelicerae with two promarginal and three retromarginal teeth. Leg measurements: I 2.46 (0.72, 0.22, 0.56, 0.51, 0.45); II 2.38 (0.70, 0.21, 0.53, 0.50, 0.44); III 2.27 (0.64, 0.20, 0.50, 0.50, 0.43); IV 2.94 (0.80, 0.23, 0.71, 0.69, 0.51). Leg spination: patella IV p1; tibiae III–IV p1 r1 v1.

***Coloration*** (Figs 28G, 29G). Carapace pale yellow, with a few long brown hairs. Fovea unobvious. Eye absent. Chelicerae, labium, and gnathocoxae pale yellow, with long brown hairs. Sternum yellowish. Legs white. Opisthosoma oval, white. Spinnerets white.

***Epigyne*** (Fig. 27A, B). Epigynal plate as long as wide. Depression unobvious. Copulatory openings arc-shaped. Copulatory ducts long and strongly convoluted, with two turns, medium bifurcate. The short one connected to secondary spermathecae, the other connected to primary spermathecae, medium with glandular appendages. Secondary spermathecae oval, half wide of kidney-shaped primary spermathecae. Fertilization ducts directed at 9:00 o’clock position from spermathecae.

##### Etymology.

The specific epithet refers to the type locality; noun in apposition.

##### Distribution.

Known only from the type locality (Fig. 30).

## Supplementary Material

XML Treatment for Goblinia

XML Treatment for Goblinia tiane

XML Treatment for Myahnia

XML Treatment for Myahnia kanpetlet

XML Treatment for Troglohnia

XML Treatment for Troglohnia dafang

XML Treatment for Troglohnia qiubei

XML Treatment for Troglohnia shidian

XML Treatment for Troglohnia wuding

XML Treatment for Typhlohnia

XML Treatment for Typhlohnia banlaksao

XML Treatment for Typhlohnia kaiyang

XML Treatment for Typhlohnia rongshui

XML Treatment for Typhlohnia sondoong

XML Treatment for Typhlohnia suiyang

## ﻿Additional information

### Author ORCIDs

Chang Chu https://orcid.org/0000-0003-3520-5463

Yejie Lin https://orcid.org/0000-0002-6789-2731

Shuqiang Li https://orcid.org/0000-0002-3290-5416

### Data availability

All of the data that support the findings of this study are available in the main text.
